# An Osteoconductive Janus Hydrogel with Full Barrier Protection and Adaptable Degradation Properties for Superior Bone Regeneration

**DOI:** 10.1002/advs.202506736

**Published:** 2025-06-23

**Authors:** Yanhui Lu, Jia Song, Yongle Lv, Boon Chin Heng, Mingming Xu, Ying He, Youde Liang, Lu‐Ning Wang, Tingting Wu, Ting Song, Tingjun Li, Qiaomei Ren, Lei Wang, Xuliang Deng, Xuehui Zhang

**Affiliations:** ^1^ Department of Dental Materials & Dental Medical Devices Testing Center Peking University School and Hospital of Stomatology Beijing 100081 P. R. China; ^2^ Department of Geriatric Dentistry Peking University School and Hospital of Stomatology Beijing 100081 P. R. China; ^3^ Beijing Advanced Innovation Center for Materials Genome Engineering State Key Laboratory for Advanced Metals and Materials School of Materials Science and Engineering University of Science and Technology Beijing Beijing 100083 P. R. China; ^4^ National Center for Stomatology National Clinical Research Center for Oral Diseases National Engineering Research Center of Oral Biomaterials and Digital Medical Devices NMPA Key Laboratory for Dental Materials Beijing Laboratory of Biomedical Materials & Beijing Key Laboratory of Digital Stomatology NHC Key Laboratory of Digital Stomatology Peking University School and Hospital of Stomatology Beijing 100081 P. R. China; ^5^ Oral Translational Medicine Research Center Joint Training base for Shanxi Provincial Key Laboratory in Oral and Maxillofacial Repair Reconstruction and Regeneration The First People's Hospital of Jinzhong Jinzhong Shanxi Province 030600 P. R. China; ^6^ The department of stomatology center The People's Hospital of Baoan Shenzhen 518100 P. R. China

**Keywords:** adaptable degradation, integrated Janus hydrogel, osteoconductive, protective barrier

## Abstract

Implant materials for bone regeneration necessitate a barrier function to block bacterial adhesion and fibroblast infiltration, while maintaining a delicate equilibrium between material degradation and osteogenesis. Here, a spatiotemporally and hierarchically‐guided bone regeneration hydrogel with a Janus structure is engineered through a sequential photocuring protocol, which features full barrier protection by the outer dense phase and superior osteoconductivity within the inner loose phase. The Janus hydrogel exhibits stable spatiotemporal layering, adaptable degradation, asymmetrical combination of network structures, and mechanical strength. The dense phase, with space maintenance capacity, completely covers the defective area, continuously blocking fibroblast infiltration, and preventing bacterial adhesion. In addition, the loose phase is shape‐adapted to the defective cavity, allowing osteoblast‐associated cells to migrate and create a favorable osteogenic microenvironment. In situ implantation of this Janus hydrogel effectively promoted osteogenesis, angiogenesis, and neurogenesis in both mouse calvarial and rat periodontal bone defect models. Furthermore, the osteogenic efficiency achieved by the Janus hydrogel implanted in mouse calvarial defects and rat periodontal defects is increased by 42% and 13.7%, respectively, as compared with previous studies. These findings thus demonstrated the synergy of protective barrier function, osteoconductive properties, and adaptive degradation within a single scaffold, which is conducive to bone regeneration.

## Graphical abstract

The filler‐barrier hydrogel system developed by dual‐network Janus structural strategy breaks through the difficulty of a single hydrogel to combine barrier protection with bone regeneration, and realizes the regeneration‐material degradation adaptation for the field of mandibular regeneration. Multi‐omics analysis elucidates the underlying mechanisms by which the material regulates tissue regeneration and lipid metabolism pathway.

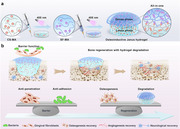

## 1 Introduction

The goal of bone regenerative medicine is to restore the normal physiological activities and functions of injured or diseased bone tissues. The optimal strategy for implant biomaterials to achieve this requires maintaining the delicate balance between material degradation and tissue regrowth to maximize material function during bone repair^[^ ^1^ ^]^ However, most studies often only consider the biological function of the implant material itself to promote bone regeneration. The infiltration of non‐osteogenic cells and tissues into the bone defect will upset the degradative–regenerative balance.^[^ ^2^, ^3^ ^]^ If bone tissue is infected, the bacterial invasion will cause further complications, which could result in failed repair or delayed healing.^[^ ^4^, ^5^ ^]^ Hence, precise coupling of different or even contradictory material properties and biological characteristics, such as barrier protection and guided regeneration, as well as preventing bacterial adhesion and promoting cell‐activation, is much desired in the field of bone tissue engineering but remains challenging to achieve.

Hydrogels have been widely used in tissue regeneration due to their advantages such as, amenability to on‐demand injection, in situ gelation, and minimally invasive implantation.^[^ ^6^, ^7^ ^]^ Moreover, hydrogels can also provide a conducive physical microenvironment for cell growth and differentiation due to their suitable network structure.^[^ ^8^, ^9^ ^]^ In recent years, injectable hydrogels with in situ gel‐forming properties upon exposure to chemical, temperature, pH or light stimuli were designed to simulate the anisotropic functional and structural characteristics of bone and have achieved progressive results in promoting osteogenesis, thereby attracting extensive attention among orthopedic researchers.^[^ ^10^, ^11^ ^]^ Jiang et al. developed a novel injectable in situ forming composite hydrogel system with physico‐chemical properties and excellent osteogenic/angiogenic function by introducing fibroin and sodium alginate for minimally invasive treatment of jaw regeneration.^[^ ^12^ ^]^ It was reported that a co‐assembly system that integrates hyaluronic acid tyramine, bioactive peptide amphiphiles, and Laponite to engineer hydrogels can be fine‐tuned to enhance bone regeneration.^[^ ^13^ ^]^ However, currently available hydrogels are limited in clinical application for bone regeneration under physiological conditions, and in particular, lack the ability of resistance to fibrous tissue infiltration and bacterial adhesion and adaptable degradation. Therefore, it is necessary to develop an advanced hydrogel‐based scaffold with distinct functionalities on opposite sides, referred to as a Janus hydrogel‐based scaffold to meet the needs of bone repair under both physiological and pathological conditions.

**Scheme 1:**
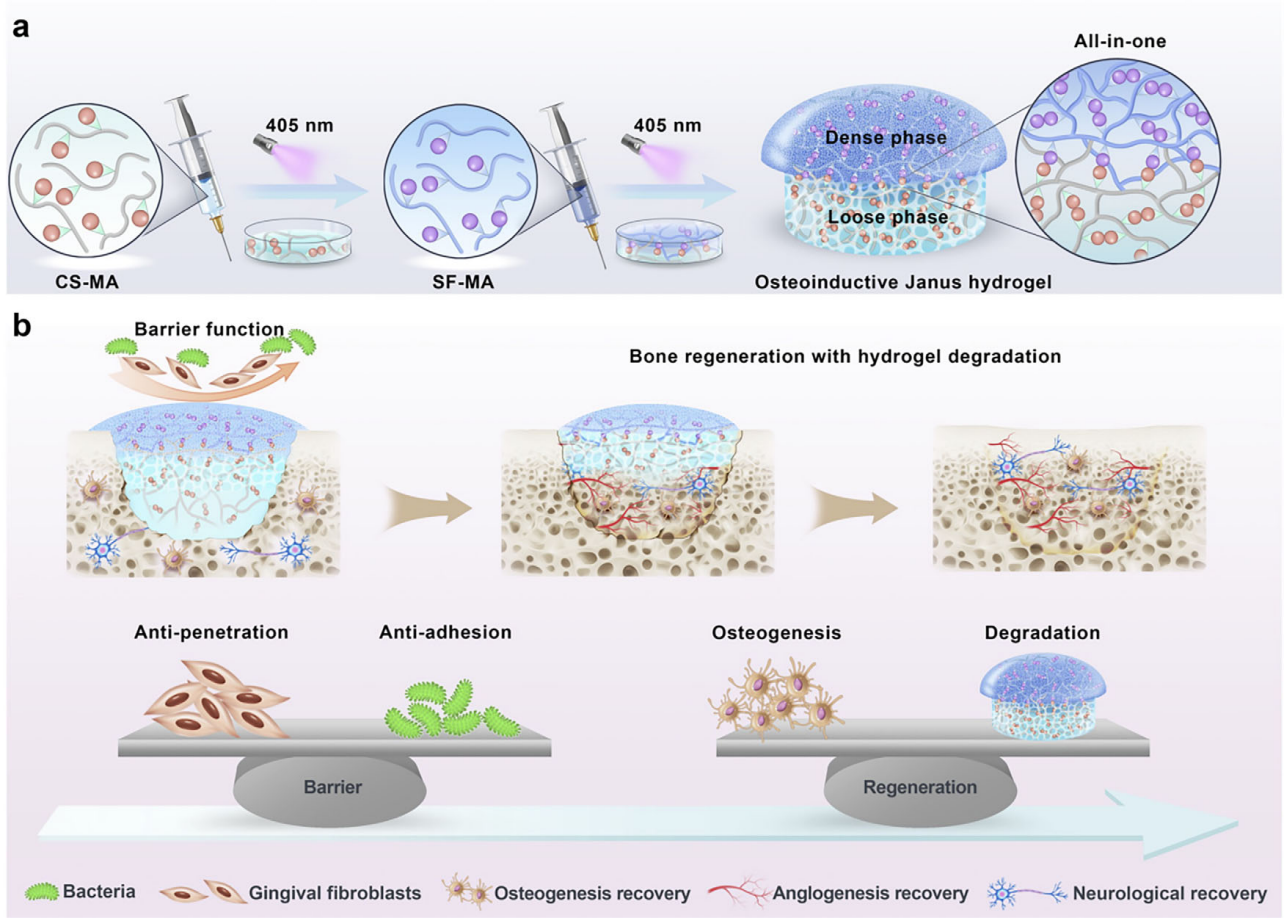
Schematic diagram of the integrated osteoconductive Janus hydrogel in bone regeneration. a) This integrated osteoconductive Janus hydrogel was fabricated by sequential application of photocuring, comprising methacrylated silk fibroin (SF‐MA) hydrogel with barrier protection properties and methacrylated chondroitin sulfate (CS‐MA) hydrogel with osteoconductive property. b) After implantation to fill the bone defect, the integrated osteoconductive Janus hydrogel facilitated improved new bone formation with adaptable degradation.

Silk fibroin (SF) and chondroitin sulfate (CS) are biocompatible and have been approved by the Food and Drug Administration (FDA) for biomedical applications.^[^ ^14^, ^15^ ^]^ Here, based on multiple adaptations including mechanics and pore structure, a spatiotemporally Janus hydrogel through a simple sequential photocuring was developed, which can comprehensively overcome various challenging issues during the regeneration processes (**Scheme** **1**). The silk‐methacrylate (SF‐MA) phase exhibits a microporous structure and adhesion to seal the edge of the defect cavity, so it has a sufficient space maintenance capacity. The chondroitin sulfate‐methacrylate (CS‐MA) phase exhibits a macroporous structure and shape plasticity that can adapt to various bone surface morphologies. Based on the above asymmetric design, we integrate the framework with plasticity to meet the desirable space properties of the regeneration proccess. In terms of the hierarchical function, the SF‐MA dense phase can continuously act as a barrier against both fibroblasts and bacteria, while the CS‐MA phase is conducive to the ingrowth of osteogenesis‐associated cells. Furthermore, we demonstrated that this integrated osteoconductive Janus hydrogel achieved adaptive degradation with new bone formation in both mouse calvarial defect and rat periodontal defect models. Mechanistic study revealed that various signaling pathways associated with cell proliferation, differentiation, and tissue regeneration were significantly activated during bone regeneration, with lipid metabolism being observed to be the most enriched among multiple metabolic pathways. Combined LC‐MS/MS analysis suggested that the integrated Janus hydrogel orchestrated cellular lipid metabolism that promoted the osteogenic differentiation of Rat Bone Marrow Mesenchymal Stem Cells (rBMSCs). Based on our results, our integrated Janus hydrogel scaffold achieves both barrier protection and osteogenic enhancement, which offers much promise in clinical bone regeneration.

## 2 Results and Discussion

### 2.1 Design Principles and Application Scenarios of the Integrated Osteoconductive Janus Hydrogel Scaffold

**Figure 1:**
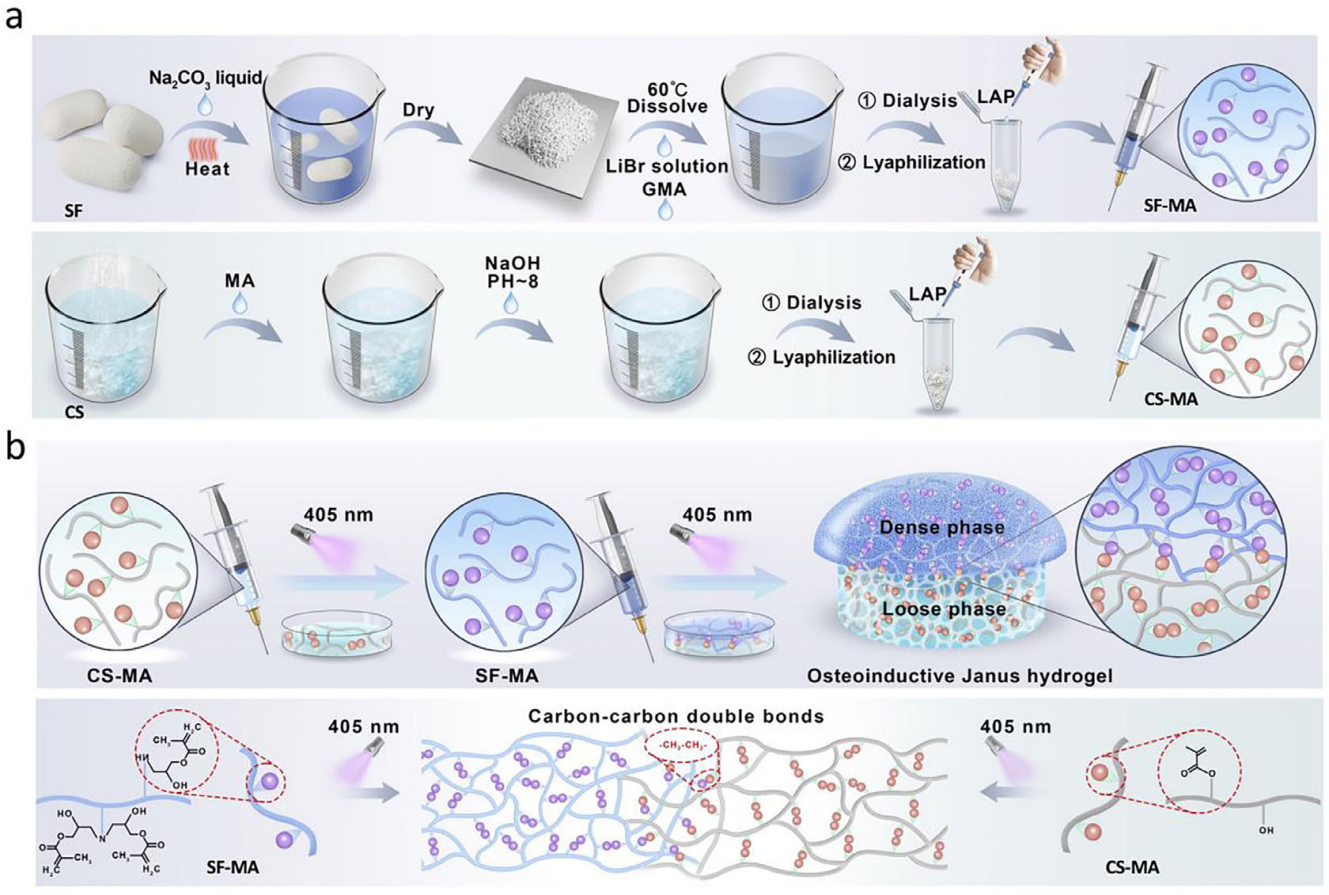
Design principles and application scenarios of the integrated osteoconductive Janus hydrogel. a) Schematic diagram of the fabrication process of the SF‐MA and CS‐MA phases. b) The design principles of the integrated osteoconductive Janus hydrogel.

Functional chemical modifications of injectable hydrogels by grafting groups or ions, is a reliable method for regulating the physical properties of injectable hydrogels.^[^ ^16^ ^]^ To realize the integration of osteoconductive and protective barrier functions for synergistically promoting bone defect repair, an integrated osteoconductive Janus hydrogel was fabricated, which was composed of SF‐MA hydrogel and CS‐MA hydrogel. Previous studies have shown that CS is a biologically active polysaccharide that enhances the expression of osteogenic genes and repair of bone microstructure.^[^ ^17^ ^]^ Moreover, CS polymer chains contain a number of sulfate and carboxyl groups that regulate cytokine recruitment and promote cell adhesion, migration, proliferation, and differentiation.^[^ ^18^, ^19^ ^]^ SF is a natural fiber polymer extracted for wound dressings, cartilage regeneration, and other tissue engineering because of its biocompatibility, biodegradability, and high strength.^[^ ^20^, ^21^, ^22^ ^]^ SF can also be used as an important material source for guiding bone regeneration membranes in bone tissue reconstruction.^[^ ^23^, ^24^ ^]^ Additionally, a previous study demonstrated that owing to the presence of carboxyl groups within the amorphous region of the SF molecular chains, the SF coating increased the polarity and water contact angle of the materials.^[^ ^25^ ^]^ The successful introduction of carbon–carbon double bonds into the molecular chains of CS and SF through chemical modification techniques achieves the synthesis of photocurable hydrogel (CS‐MA and SF‐MA) (**Figure** **1**a). Due to the presence of gingival fibroblasts and the high microbial density of the oral environment, periodontal defect healing often has a poor prognosis. To overcome the aforementioned clinical challenges, the integrated osteoconductive Janus hydrogel was obtained by sequential injection and photocuring (Figure 1b). The chemical structures of CS‐MA and SF‐MA were confirmed by ^1^H NMR spectrum (Figure S1a, Supporting Information), which clearly exhibited the signals of vinyl protons at 5.6 and 6.2 ppm (─CH_2_). In this study, the CS‐MA hydrogel was designed with two concentrations of 5% w/v (LCS) and 10% w/v (HCS), which was aimed at investigating the effects of bonding and regulating cytokines and growth factors involved in osteogenesis. For the SF‐MA hydrogel, we designed three hydrogels with concentrations of 5% w/v (SF‐MA 5%), 10% w/v (SF‐MA 10%), and 30% w/v (SF‐MA) respectively. When the concentration was 30% w/v, the cross‐section of the SF‐MA hydrogel exhibited a microporous structure with a smooth and dense outer surface (Figure S1b,c, Supporting Information), as observed under scanning electron microscopy. Therefore, we chose 30% (w/v) SF‐MA hydrogel for one phase of the integrated osteoconductive Janus hydrogel in subsequent experiments.

### 2.2 Structural and Mechanical Characteristics of the Integrated Osteoconductive Janus Hydrogel

**Figure 2:**
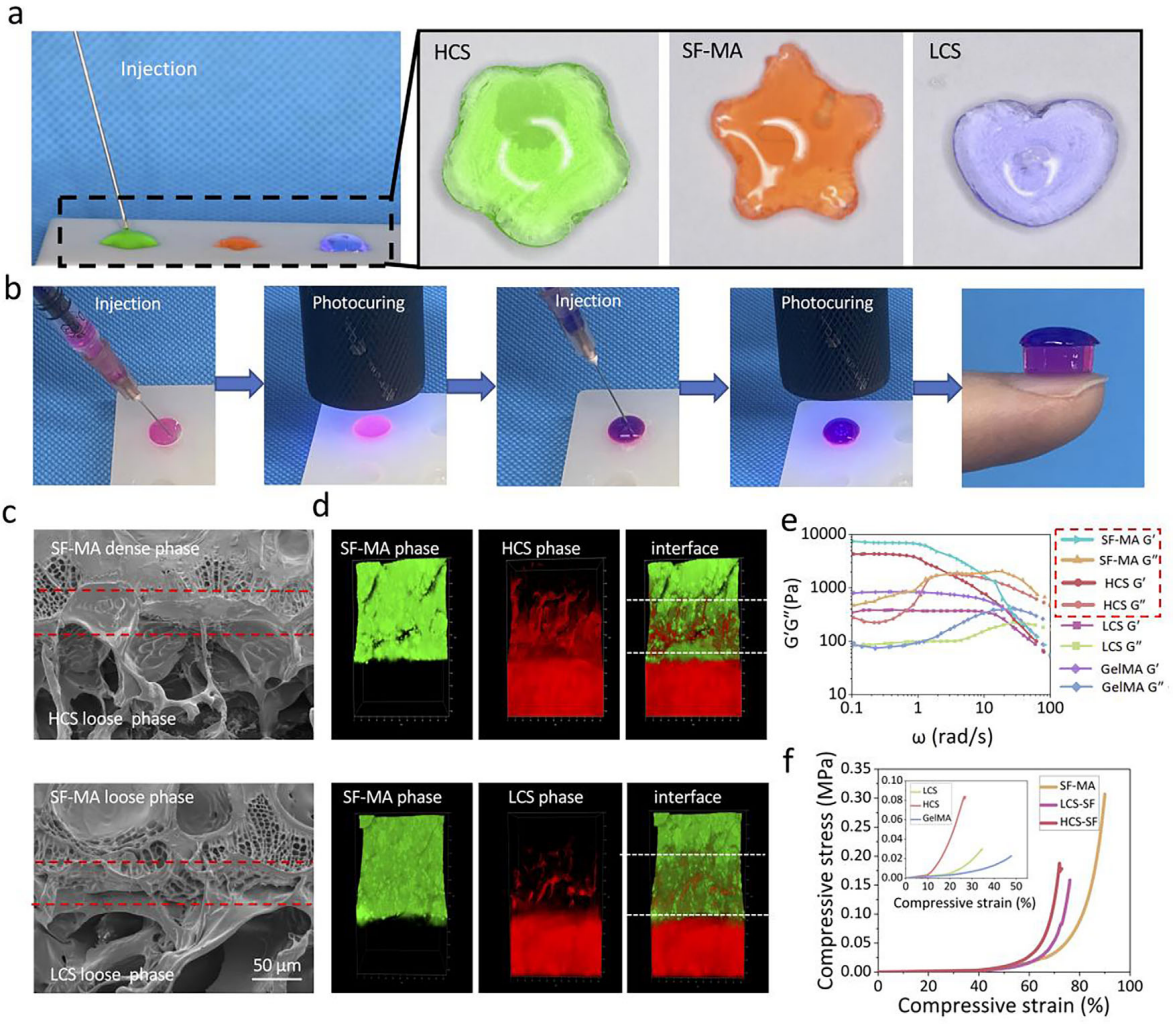
Structural and mechanical characterization of the integrated osteoconductive Janus hydrogel. a) The injectability and in‐situ gelation of the integrated osteoconductive Janus hydrogel. b) The fabrication process of the integrated osteoconductive Janus hydrogel. c) Representative SEM images of the integrated osteoconductive Janus hydrogel. The area between the red dotted lines denotes the fusion of the CS‐MA hydrogel phase with the SF‐MA hydrogel phase. d) Representative CLSM images of the integrated osteoconductive Janus hydrogel. The area between the white dotted lines shows the fusion of the CS‐MA hydrogel phase and SF‐MA hydrogel phase. e) Rheological properties of the hydrogels after photocuring as measured by the frequency sweep test at a constant strain of 1% at 25 °C. f) Stress‐strain curve of the integrated osteoconductive Janus hydrogel.

Owing to the introduction of carbon–carbon double bonds, the hydrogel exhibited the properties of injectability and rapid photocuring, which can be adjusted by irradiation time, thus enabling convenient application. As shown in **Figures** **2**a and S1d (Supporting Information), the precursor solution was able to pass through a 26‐gauge needle and displayed quick curing in molds with various shapes upon irradiation. By sequentially adding CS‐MA precursor solution to SF‐MA precursor solution and photocuring respectively, the integrated osteoconductive Janus hydrogel was thus fabricated (Figure 2b), with the SF‐MA precursor solution displaying fluidity, and being able to partially penetrate the CS‐MA hydrogel, to form a mechanical interlock with chemical bonding after photocuring. To investigate the internal structure of the integrated osteoconductive Janus hydrogel and the interface bonding between the SF‐MA hydrogel phase and CS‐MA hydrogel phase, we found that the LCS hydrogel and the HCS hydrogel had a homogeneous, porous structure, with the pore size of the LCS hydrogel being 102.78±2.13 µm and that of the HCS hydrogel being 66.72±2.01 µm. By contrast, the structure of the SF‐MA hydrogel was relatively microporous with a pore size of just 5.06±0.19 µm (Figure 2c; Figure S1e, Supporting Information). Additionally, the SF‐MA hydrogel and the CS‐MA hydrogel were closely integrated with no obvious gap being observed. Longitudinal‐sectional scanning showed a well‐integrated interface between the SF‐MA hydrogel phase and the CS‐MA hydrogel phase, with the SF‐MA hydrogel penetrating the CS‐MA hydrogel such that mechanical interlocking and cohesion could be observed, which further confirmed the physical integrity of the integrated osteoconductive Janus hydrogel (Figure 2d). Sufficient mechanical strength and stability of the hydrogel are very important for functional stability and for creating a conducive pro‐osteogenic microenvironment.^[^ ^26^, ^27^ ^]^ As shown in Figure 2e, all hydrogels exhibited similar non‐linear rheological behavior. In the angular frequency range (0.1–10 rad s^−1^), the storage modulus (G') is higher than the loss modulus (G''). This indicated that all hydrogels had stable 3D network structures, in which they could be able to maintain the original hydrogel network structure during the functional state. Hydrogels are thought to better mimic the natural bone ECM in complex bone defects microenvironments, providing mechanical signals to promote cell adhesion, proliferation, and osteogenic differentiation.^[^ ^28^ ^]^ The results in Figure 2f and Figure S1f,g (Supporting Information) showed that the contents of CS‐MA in the hydrogel matrix exerted a significant effect on the rheological and mechanical properties of the hydrogel. With an increase of CS‐MA content, the mechanical properties of the integrated osteoconductive Janus hydrogel were improved, which could be attributed to their higher cross‐linking density. Moreover, the SF‐MA hydrogel showed stronger mechanical properties compared with the CS‐MA hydrogel or the GelMA (Control). The mechanical strength of our hydrogels greatly exceeded that of the natural hematoma fibrin clot, which was regarded as the minimum strength during the bone healing process.^[^ ^29^, ^30^ ^]^ These results suggested that the Janus hydrogel met the requirements in the complex mechanical environment of bone defect sites. Together, we successfully fabricated an integrated osteoconductive Janus hydrogel, which exhibited a stable 3D network structure and considerable mechanical properties.

### 2.3 In Vitro Swelling Behavior, Degradation Performance and Structural Stability

**Figure 3:**
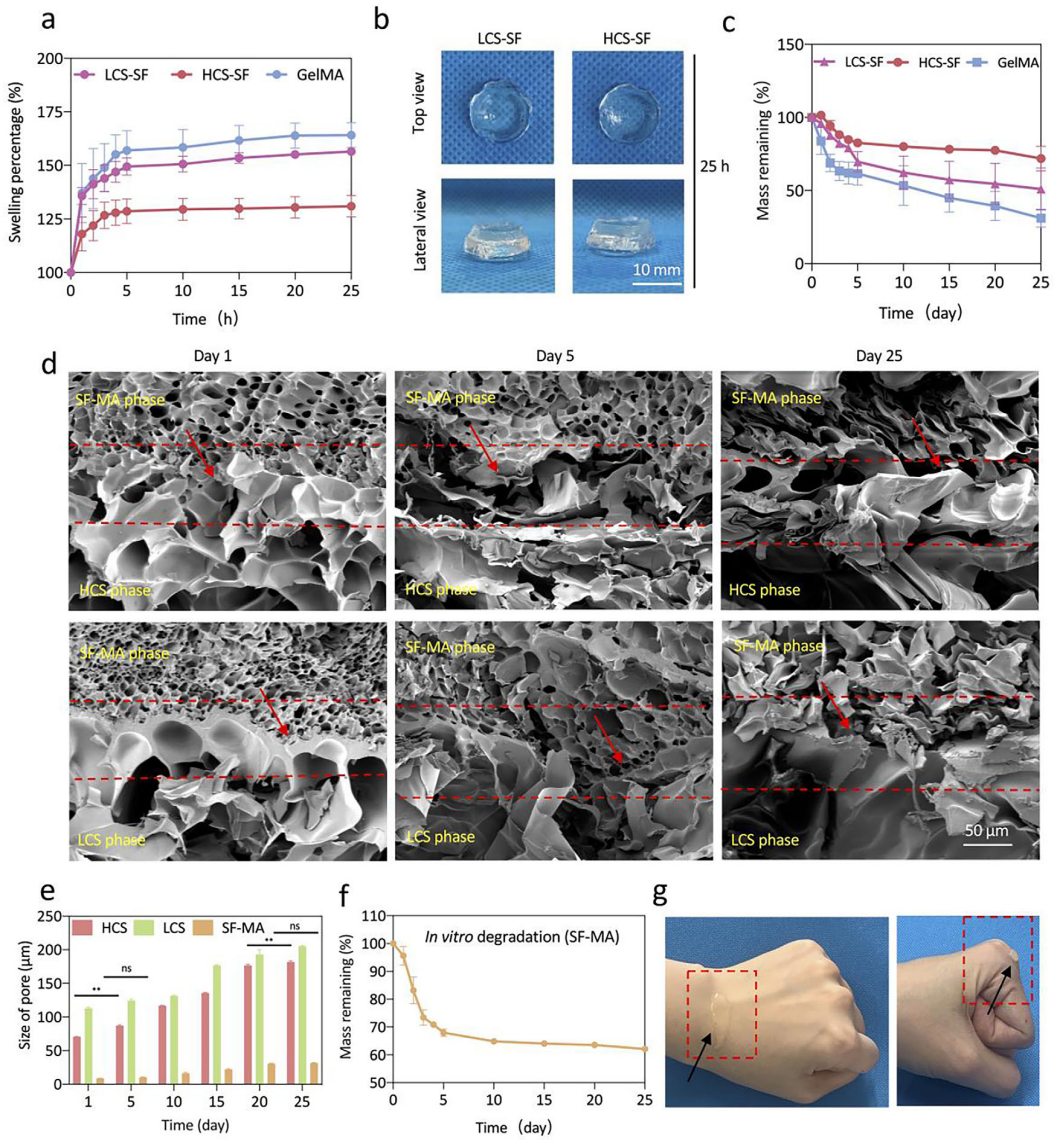
The integrated osteoconductive Janus hydrogel maintained a relatively stable structure in vitro. a) Swelling ratio of the integrated osteoconductive Janus hydrogel in PBS solution at 37 °C for 25 h (*n* = 3). b) Representative real‐time images of the integrated osteoconductive Janus hydrogel swelling over 25 h. c) Degradation of the integrated osteoconductive Janus hydrogel in vitro (*n* = 3). d) Representative SEM images after 1, 5 and 15 days of hydrogel degradation. The area between the red dotted lines denoted the fusion of the CS‐MA hydrogel phase with the SF‐MA hydrogel phase. e) Quantitative analysis of pore size within the integrated osteoconductive Janus hydrogel after degradation. f) In vitro degradation properties of the SF‐MA hydrogel. g) Tissue adhesion of the SF‐MA hydrogel. The black arrow indicated that the SF‐MA adhered to the tissue. (ns, not significant; ^***^ *p* < 0.001 and ^****^ *p* < 0.0001).

The swelling properties of the hydrogel within the in vivo environment affect its function.^[^ ^31^, ^32^ ^]^ Hydrogels in contact with blood will rapidly swell without dissolving in vivo, which can mimic the natural tissue environment to provide support for the defects. However, too much swelling performance can lead to its original function and structure being affected. By evaluating the swelling behavior of the integrated osteoconductive Janus hydrogel within modified simulated body fluid (SBF), we found that both the LCS‐SF‐MA (LCS‐SF) hydrogel and the HCS‐SF‐MA (HCS‐SF) integrated osteoconductive Janus hydrogel rapidly swelled during the 1 h, and gradually reached swelling equilibrium after 5 h (**Figure** **3**a; Figure S2a, Supporting Information). Hydrogels with high concentrations have been reported to exhibit higher cross‐linking densities due to having more carbon–carbon double bonds.^[^ ^33^ ^]^ As expected, the HCS‐SF integrated osteoconductive Janus hydrogel displayed less swelling capacity with an equilibrium swelling percentage of 120% compared to the LCS‐SF integrated osteoconductive Janus hydrogel, which was mainly attributed to its higher concentration of CS (Figure 3a). Notably, both the LCS‐SF and HCS‐SF integrated Janus hydrogel stably maintained the combination during the swelling process, which proved the organic binding of the two phases (Figure 3b). The degradation performance of the hydrogels in vitro was conducted in SBF over 25 days to mimic degradation in the biosystem.^[^ ^34^, ^35^ ^]^ Upon exposure to SBF, all the hydrogels displayed partial degradation behavior in the first 5 days with different degradation rates. The GelMA hydrogel showed the fastest degradation rate while the HCS‐SF integrated osteoconductive Janus hydrogel exhibited the slowest degradation rate (≈72% remaining after 25 days), which was also attributed to light‐induced crosslinking of carbon–carbon double bonds and high concentrations of CS (Figure 3c). Moreover, it was revealed that the integrated osteoconductive Janus hydrogel interfacial bonding remained stable during the 25‐day in vitro degradation process. With the prolongation of degradation time, the pores of the CS‐MA hydrogel gradually increased and the structure disintegrated, while the SF‐MA hydrogel still maintained a stable structure when the pores increased (Figure 3d; Figure S2b, Supporting Information). Notably, the average pore size of the SF‐MA hydrogel was still less than 35 µm on the 25^th^ day of degradation (Figure 3e). Additionally, we also found that the in vitro weight loss of the SF‐MA hydrogel was only 30% on the 25^th^ day, while the in vivo weight loss of SF‐MA hydrogel was only 50% on the 35^th^ day (Figure 3f; Figure S2c, Supporting Information). As shown in Figure 3g, we observed the adhesion of SF‐MA hydrogel to the tissue. Early removal of biological barrier membrane or premature loss of barrier structure due to rapid absorption will lead to poor osteogenesis.^[^ ^36^, ^37^ ^]^ The above results demonstrated that the integrated osteoconductive Janus hydrogels have a relatively stable structure in SBF solution with rapid swelling equilibrium and appropriate degradation rate adapted to the needs of neo‐natal bone repair, which are critical for bone regeneration.

### 2.4 In Vitro Assessment of the Barrier Protection Functions of the SF‐MA Hydrogel Phase and In Vitro Assessment of the Pro‐Osteogenic Activity of the CS‐MA Hydrogel Phase

**Figure 4:**
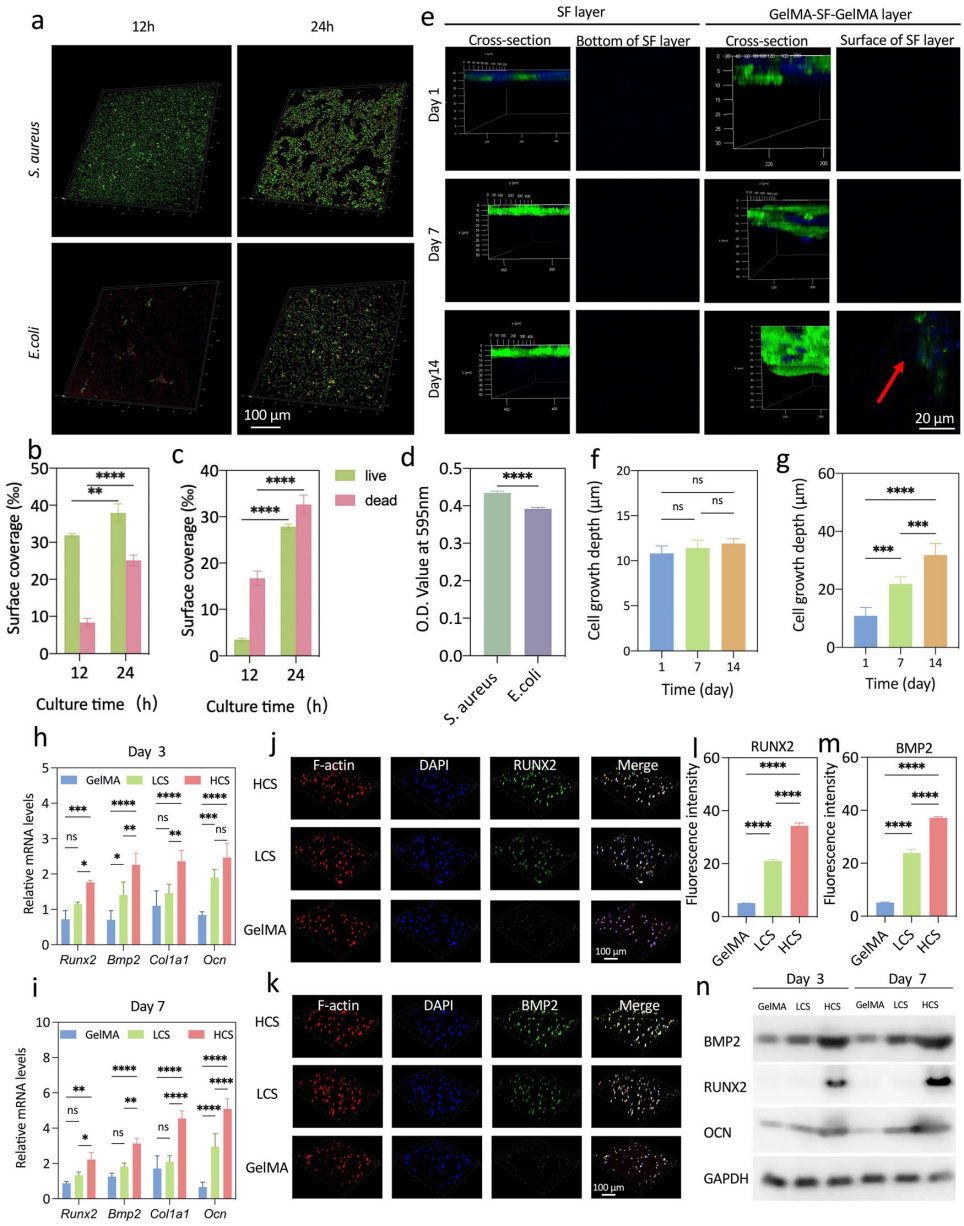
In vitro barrier protection functions of the SF‐MA hydrogel phase and in vitro pro‐osteogenic activity of the CS‐MA hydrogel phase. a) The representative live & dead staining images of *S. aureus* and *E.coli* after 24 h of co‐culture with SF‐MA. b) Quantification of the fluorescence area based on the live/dead fluorescence staining images of *S. aureus*. c) Quantification of the fluorescence area based on the live/dead fluorescence staining images of *E.coli*. d) Semi‐quantification of crystal violet staining after 48 h of co‐culture with *S. aureus* and *E.coli*. e) Representative immunocytochemical staining images of gingival fibroblasts cultured on the SF hydrogel and GelMA‐SF‐MA‐GelMA sandwich structure for 1, 7 and 14 days. The red arrow indicated that the gingival fibroblasts penetrated through GelMA to the surface of the SF hydrogel. f,g) Quantitative analysis of the growth depth of gingival fibroblasts cultured on the. SF hydrogel and GelMA‐SF‐MA‐ GelMA sandwich structure for 1, 7 and 14 days (n = 3). h) RT‐qPCR quantification of genes related to osteogenic differentiation (Runx2, Bmp2 and Ocn, Col1al) in rBMSCs cultured on LCS, HCS and GelMA for 3 days (n = 3). i) RT‐qPCR quantification of genes related to osteogenic differentiation (Runx2, Bmp2 and Ocn, Col1al) in rBMSCs cultured on LCS, HCS and GelMA for 7 days (n = 3). j) Representative immunofluorescence images of osteogenic differentiation protein (RUNX2 green), actin network (Phalloidin, red), and cell nuclei (DAPI, blue) in rBMSCs cultured on the LCS and HCS for 3 days. k) Representative immunocytochemical staining images of osteogenic marker protein (BMP2 green), actin network (Phalloidin, red), and cell nuclei (DAPI, blue) in rBMSCs cultured on the LCS and HCS for 7 days. l,m) The mean fluorescence intensities were calculated to evaluate protein (RUNX2 and BMP2) expression levels (n = 3). n) Western blot analysis of RUNX2, BMP2 and OCN in rBMSCs cultured on LCS, HCS and GelMA for 7 days. Error bars represent the standard error of the mean. (ns, not significant; ^*^ *p* < 0.05, ^**^ *p* < 0.01, ^***^ *p* < 0.001 and ^****^ *p* < 0.0001).

Figure S3a,b (Supporting Information) showed that the SF‐MA hydrogel phase was mildly hydrophobic, which made it difficult for bacteria and cells to form local adhesion sites, as the air layer formed on the surface kept out bacteria.^[^ ^38^, ^39^ ^]^ Subsequently, we measured the surface zeta potential of the SF‐MA hydrogel. We found that the surface potential of the SF‐MA hydrogel decreased significantly with increasing concentration (Figure S3c, Supporting Information), factors that are unfavorable for bacterial adhesion. It was reported that SF has low immunogenicity and causes mild inflammation during the initial stages of trauma repair, favoring the destruction of pathogens present at the site of injury.^[^ ^40^ ^]^ As the predominant etiological microbe implicated in oral infections, *Staphylococcus aureus (S. aureus)* and *Escherichia coli (E.coli)* were selected for in vitro experiments. The results showed that the activity and proliferation of both *S. aureus* and *E. coli* were inhibited with decreasing surface zeta potential when co‐cultured with the SF‐MA hydrogel (Figure S3d,e, Supporting Information). As shown in **Figures** **4**a and S3f (Supporting Information), the bacterial colony‐forming units of *S. aureus* and *E. coli* decreased with decreasing surface potential, as assessed by confocal laser scanning microscope (CLSM). With the extension of co‐culture time, the number of bacteria on SF‐MA increased, but it must be noted that the proportion of dead bacteria also increased (Figure 4b,c; Figure S3g,h, Supporting Information). Biofilm formation is a key mechanism for bacteria to resist harsh environments and enable drug resistance.^[^ ^41^ ^]^ We therefore assessed biofilm formation by crystal violet staining and the same trend was confirmed (Figure 4d; Figure S3i,j, Supporting Information). In brief, the above results suggested that the dense SF phase was effective in resisting bacterial adhesion. To verify the feasibility of in vivo applications of the integrated osteoconductive Janus hydrogel, we first conducted biocompatibility assays by culturing rBMSCs in the CS‐MA hydrogel and human gingival fibroblasts (HGFs) in the SF‐MA hydrogel. The cell viability was assessed by CCK‐8 assay and the results showed that all groups of hydrogels exhibited biocompatibility, with rBMSCs in the CS‐MA hydrogels and HGFs in the SF‐MA hydrogel proliferating normally when cultured for 1, 3, 7 and 14 days (Figure S4a, Supporting Information). Moreover, for clinical GBR treatment, a protective barrier to prevent fibroblasts infiltration is of great significance for occupying the bone defect space and preventing fibroblast infiltration from the surrounding soft tissue.^[^ ^42^, ^43^, ^44^ ^]^ Considering the mechanical properties and microporous structure of the SF‐MA hydrogel, we next assessed the function of preventing fibroblast infiltration within a simulated in vivo environment by culturing HGFs on the surface of the SF‐MA hydrogel. The results showed that HGFs hardly penetrated the SF‐MA hydrogel with a growth depth of only 12 µm after 14 days of culture (Figure 4e,f; Figure S4b,c, Supporting Information). For comparison, we also constructed a hydrogel scaffold with a GelMA‐SF‐MA‐GelMA sandwich structure, and cultured HGFs on the surface of the GelMA hydrogel. HGFs gradually infiltrated into the GelMA hydrogel, and the depth of growth reached 38 µm after 14 days, which further proved that the SF‐MA hydrogel had the effective function of preventing fibroblasts infiltration (Figure 4g). Hence, our data demonstrated that the SF‐MA hydrogel phase exhibited a protective barrier function, which provided structural and biological protection for the bone regeneration process.

To assess the pro‐osteogenic functions of the LCS hydrogel and HCS hydrogel on rBMSCs, we co‐cultured rBMSCs with hydrogels for 3 and 7 days and measured the expression of osteogenesis‐related genes (*Col1al, Ocn, Bmp2, Runx2*) by RT‐qPCR. Compared with the control group, osteogenesis‐related genes were upregulated in cells cultured in both the LCS hydrogel and HCS hydrogel (Figure 4h,i). Notably, transcription levels of these genes were much higher in the HCS groups, thus suggesting the significant stimulatory role of high concentrations of CS within the hydrogels in promoting osteogenesis. Immunofluorescence staining (RUNX2 and BMP2) was performed to analyze the localization and quantity of osteogenic markers in rBMSCs on day 7. As demonstrated in Figure 4j,k, upregulation of RUNX2 and BMP2 was observed in both the LCS hydrogel and HCS hydrogel, within the nucleus and cytoplasm, respectively, indicating that the main switch of osteogenic differentiation was turned on, especially in the HCS hydrogel. The quantitative analyses of RUNX2 and BMP2 protein expression shown in Figure 4l,m also confirmed the trend. Western blotting further confirmed the enhancing effects of LCS and HCS on the osteogenic differentiation of rBMSCs, with upregulated expression of early osteogenic differentiation‐related markers RUNX2, BMP2, and OCN in rBMSCs after 7 days of culture (Figure 4n). Taken together, our results revealed that the CS‐MA hydrogel phase can activate osteogenic differentiation of rBMSCs in vitro.

### 2.5 Dynamic Adaptation of Material Degradation with New Bone Formation

**Figure 5:**
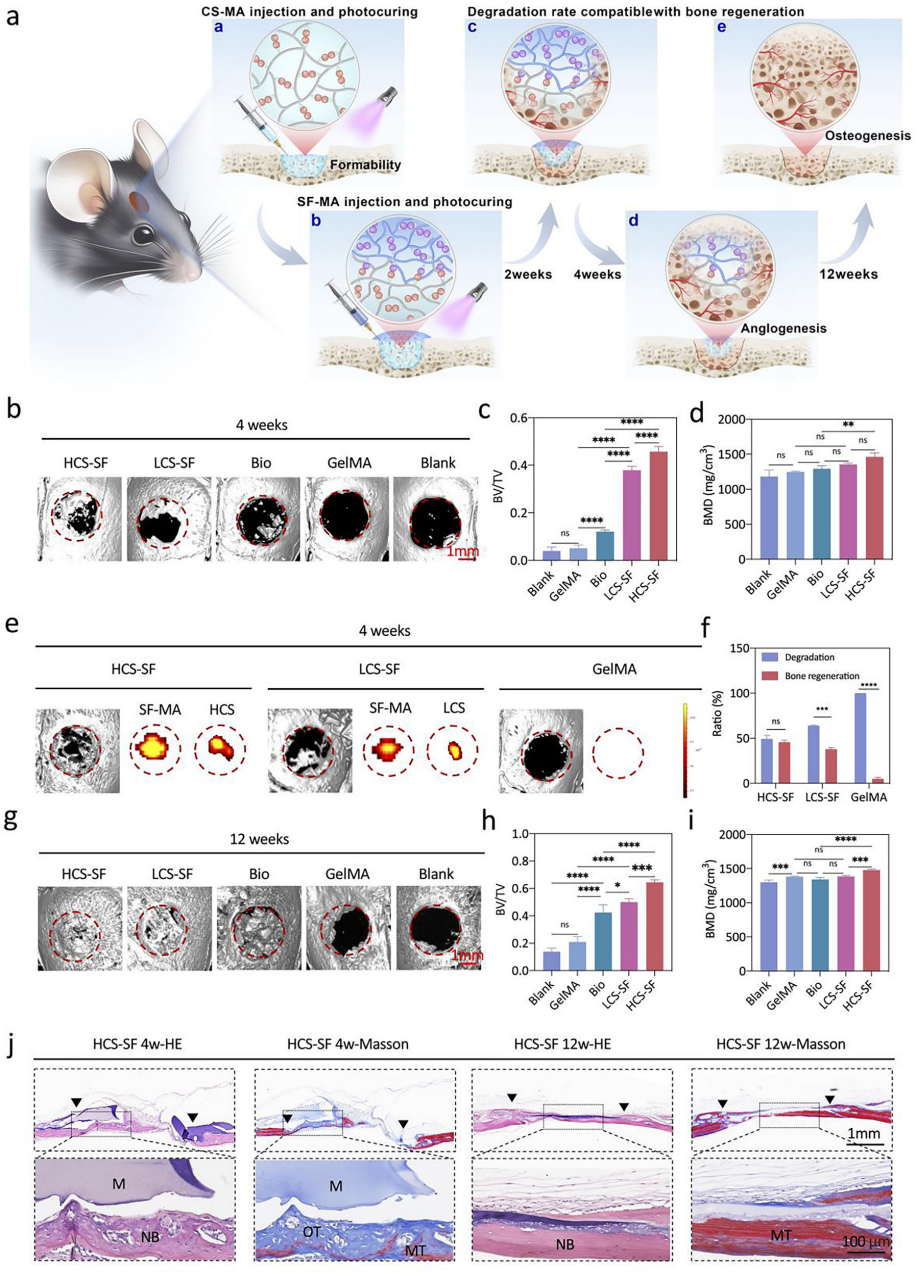
The integrated osteoconductive Janus hydrogel achieved adaptive degradation of materials with new bone formation. a) Schematic representation of adaptation between hydrogel degradation and bone regeneration within the mouse calvarial defect area. b) Representative micro‐CT images of mouse calvarial full‐thickness defects at 4 weeks post‐implantation. Red dotted lines denote the boundaries between the nascent bone and the host bone. c,d) Quantitative analysis of the bone volume/ tissue volume and bone mineral density (BMD) at 4 weeks post‐implantation (*n* = 4). e) Representative micro‐CT images of mouse calvarial full‐thickness defects at 4 weeks post‐implantation and degradation images of the integrated osteoconductive Janus hydrogel in vivo at 4 weeks post‐implantation. f) Quantification analysis of the degradation ratio of the integrated osteoconductive Janus hydrogel in vivo and new bone formation ratio at 4 weeks post‐implantation (*n* = 4). g) Representative micro‐CT images of mouse calvarial full‐thickness defects at 12 weeks post‐implantation. The red dotted lines denote the boundaries between the nascent bone and the host bone. h,i) Quantitative analysis of bone volume/ tissue volume and bone mineral density (BMD) at 12 weeks post‐implantation (*n* = 4). j) H&E staining and Masson's trichrome staining of histological sections at 4 & 12 weeks after implantation. (FT, fibrous tissue; NB, nascent bone; OT, osteoid tissue; MT, mineralized tissue; M, residual materials; P, periosteum). Error bars represent the standard error of the mean. (ns, not significant; ^*^ *p* < 0.05, ^**^ *p* < 0.01, ^***^ *p* < 0.001 and ^****^ *p* < 0.0001).

The adaptive degradation of scaffold materials with new bone formation is a key indicator in biodegradable material‐mediated bone regeneration.^[^ ^45^, ^46^, ^47^ ^]^ To further evaluate the degradation behavior of the integrated osteoconductive Janus hydrogel in vivo, we labeled the hydrogels with fluorescent dyes and implanted the scaffold into the calvarial defect of mice (**Figure** **5**a). The first stage of bone defect reconstruction is the growth of periosteum. The complete periosteum covering the defect area is helpful in preventing the ingrowth of gingival fibrous tissues, thereby preserving space for new bone generation.^[^ ^48^, ^49^ ^]^ Before the periosteum is fully repaired, the SF‐MA hydrogel phase needs to function as a barrier to protect the bone defect from soft tissue ingrowth. The SF‐MA hydrogel degraded slowly in vivo during the first 28 days, effectively protecting the bone defect area and promoting periosteal repair, followed by being gradually degraded, with more than 50% remaining at 35 days (Figure S5a,b, Supporting Information). After the implantation of the materials, the integrated osteoconductive Janus hydrogel also started to display osteogenic repair function at the same time. As shown in Figure S5b (Supporting Information), HCS‐SF and LCS‐SF were gradually degraded after implantation, and the degradation rate of the HCS‐SF integrated Janus hydrogel was slower than that of the LCS‐SF integrated Janus hydrogel, with HCS‐SF and LCS‐SF degrading by 35.8% and 49.6% on the 28th day respectively. In addition, the periosteum had formed and covered the entire defect area by 28 days, and new bone had been generated in the bone defect area, while the integrated osteoconductive Janus hydrogel had been partially degraded based on micro‐CT scanning and histological analysis (Figure S5c, Supporting Information). In contrast, the GelMA group was completely degraded by 21 days with ineffective bone regeneration (Figure S5b, Supporting Information; Figure 5b–d,j), which indicated that the integrated osteoconductive Janus hydrogel had a more stable structure and could sustain a longer‐lasting pro‐osteogenic role in vivo. Moreover, through analysis by synthesis of calvaria neogenesis and material degradation at 4 weeks post‐implantation, we found that the bone regeneration and material degradation rate matched better in the HCS‐SF group, as compared with the GelMA control group (Figure 5e,f). Although the in vivo degradation rate of materials in the LCS‐SF group was slightly faster than that of bone regeneration, it still supported bone growth during the early stages of bone regeneration. At 12 weeks post‐implantation, the defects were almost completely filled with new bone in the HCS‐SF, LCS‐SF and Bio‐Gide groups (Figure 5g). The new bone volume/total volume (BV/TV) of the group implanted with HCS‐SF was higher than the control groups (Figure 5h) at 12 weeks post‐implantation. Bone mineral density (BMD) was also measured and there was a significant difference between the HCS‐SF group and the Bio‐Gide group at 12 weeks post‐implantation (Figure 5i). These results thus suggested that the integrated osteoconductive Janus hydrogel mainly played a role in osteogenic stimulation during the early‐middle process of bone regeneration. Subsequent H&E staining and Masson's trichrome staining were used to histologically analyze the osteogenic process (Figure 5j; Figure S5d, Supporting Information). At 12 weeks post‐implantation, HCS‐SF led to complete healing with flat and consecutive bone‐structure formation that is characteristic of full bone maturation. Masson's trichrome staining also revealed mature osteoid tissue after 12 weeks of implantation of HCS‐SF. In contrast, a small amount of newly‐formed bone was observed in the GelMA control group, without any complete and contiguous healing with host tissues, with only fibrous tissues being detected in the Blank group when observation time was extended to 12 weeks post‐implantation. These results thus indicated that the coordination of the barrier function of the SF‐MA hydrogel phase and the osteoconductive function of the CS‐MA hydrogel phase together with adaptive degradation, contributed to the regeneration of the mouse calvarial defect. In summary, the integrated osteoconductive Janus hydrogel has a degradation rate compatible with bone regeneration, which promoted healing of bone defects and subsequent bone maturation.

### 2.6 The Integrated Osteoconductive Janus Hydrogel Enhanced Rat Periodontal Bone Regeneration

**Figure 6:**
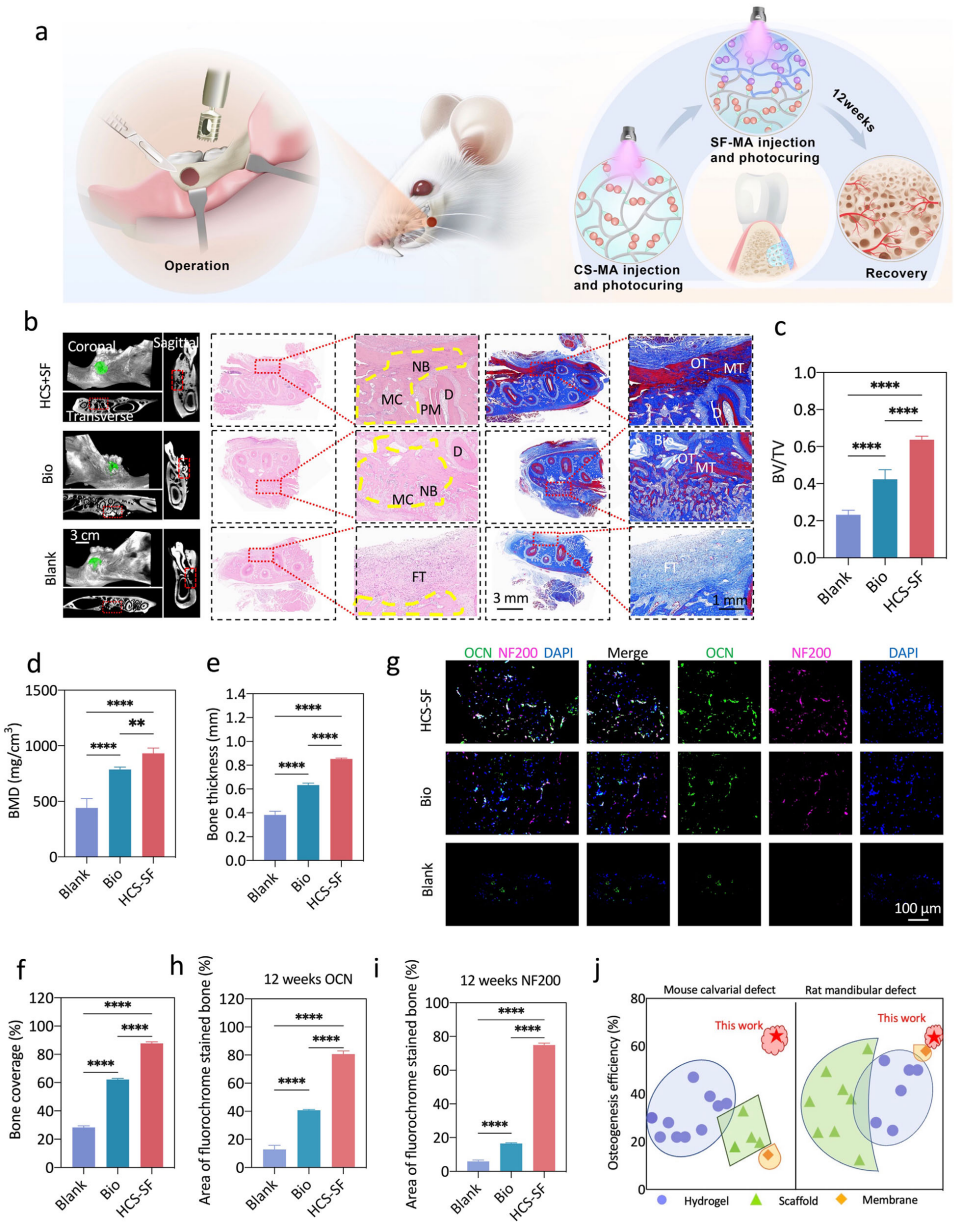
The integrated osteoconductive Janus hydrogel enhanced rat periodontal bone regeneration. a) Schematic representation of the integrated osteoconductive Janus hydrogel implantation within the rat periodontal defect area. b) Representative micro‐CT images of rat periodontal defects at 12 weeks post‐implantation. Red dotted lines denote the boundary between nascent bone and host bone. The green fake color denotes nascent bone. H&E staining and Masson's trichrome staining of histological sections at 12 weeks after post‐implantation. The parts circled by the yellow dotted line denote the new bone in the defect area. (FT, fibrous tissue; NB: nascent bone; OT, osteoid tissue; MT, mineralized tissue; MC, marrow cavity; D, dentin; PM, periodontium). c–f) Quantitative analysis of bone volume/ tissue volume, bone mineral density (BMD), bone thickness and bone coverage at 12 weeks post‐implantation (*n* = 5). g) Immunofluorescence images of OCN (Green) and NF200 (Pink) expression within the rat mandible defect after implantation of the integrated osteoconductive Janus hydrogel at 12 weeks post‐implantation. DAPI stained cell nuclei (Blue). h,i) The mean fluorescence intensities were calculated to evaluate protein expression levels (*n* = 5). j) Osteogenesis efficiency of HCS‐SF hydrogel (red asterisk), as compared with hydrogel materials (purple circles), scaffold materials (green triangles) and membrane materials (orange rhomboids) within the mouse calvarial defect model and rat periodontal defect model. Materials are classified according to the material morphology. The osteogenesis efficiency is represented by the ratio of bone volume to the total volume (BV/TV). Details and values of the aforementioned materials are listed in Table S1 (Supporting Information). Error bars represent the standard error of the mean. (ns, not significant; ^*^ *p* < 0.05, ^**^ *p* < 0.01, ^***^ *p* < 0.001 and ^****^ *p* < 0.0001).

To further investigate the osteoconductive functions of the integrated osteoconductive Janus hydrogel for clinical GBR therapy, a rat mandibular complete periodontal defect model (with a 3 mm × 1 mm‐sized defect) was established (**Figure** **6**a). Rats treated with Bio‐Gide and Bio‐Oss (Bio) were assigned as controls; while untreated rats were assigned as the Blank group. Through micro‐CT and histological analysis, we found that there was almost no new bone formation in the Blank group, at 4 weeks and even 12 weeks post‐implantation (Figure S6a, Supporting Information; Figure 6b), thus indicating the weak self‐regenerative capacity of periodontal tissue. At 4 weeks after implantation, relatively contiguous and intact newly‐formed bone in the periodontal defect area was observed in the HCS‐SF group (Figure S6a, Supporting Information). In contrast, only a small amount of new bone was formed in the Bio group with much Bio‐Oss residues (Figure S6a, Supporting Information). The results presented in Figure S6b–e (Supporting Information) indicated that the bone volume and mean thickness of the newly formed bone were significantly greater in the HCS‐SF group versus other groups. The abovementioned results thus indicated that the integrated osteoconductive Janus hydrogel could promote stem cell osteogenic differentiation, which was consistent with the in vitro cell regulation results.

**Figure 7:**
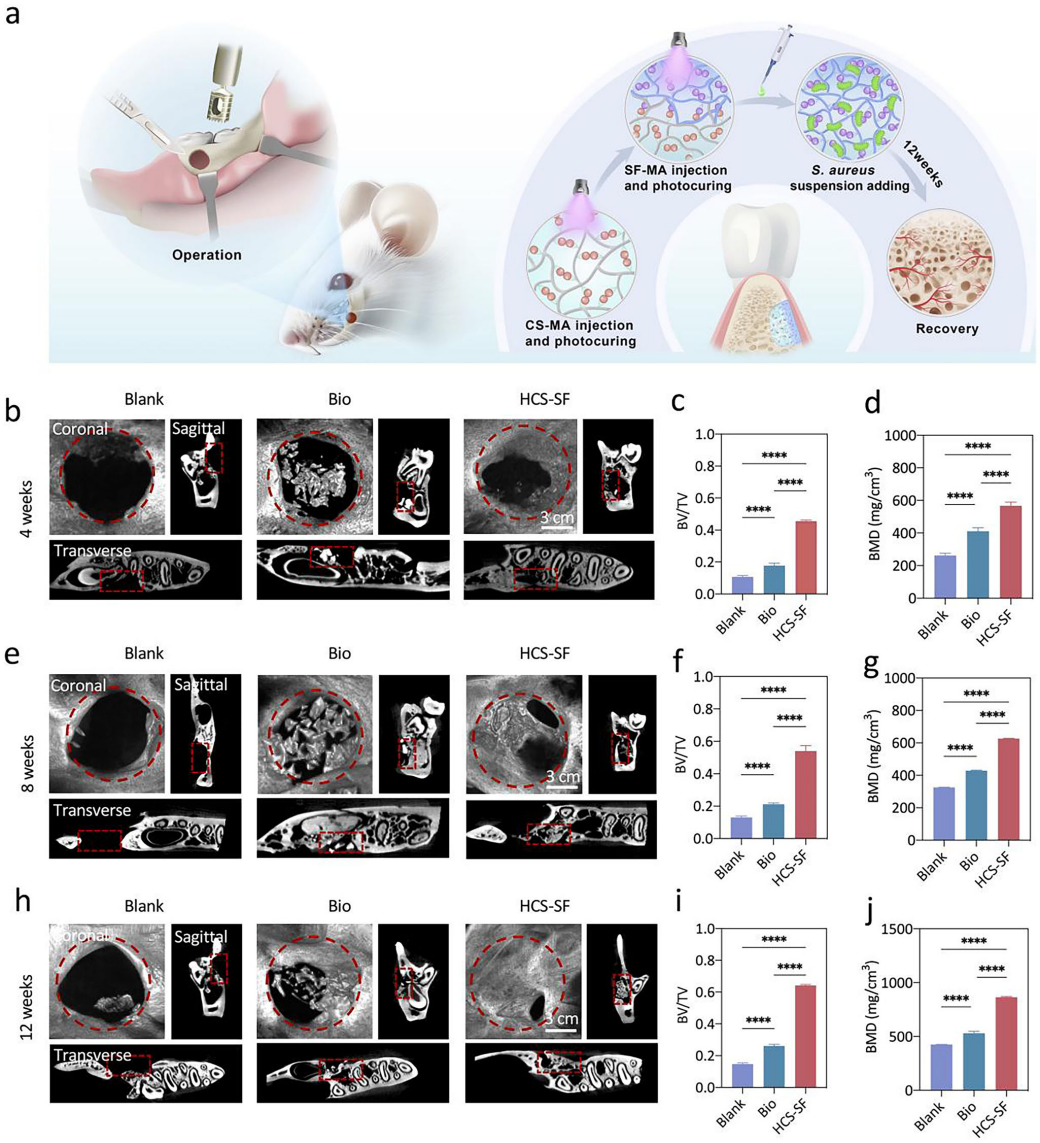
The integrated osteoconductive Janus hydrogel enhanced infected rat periodontal bone regeneration. a) Schematic representation showing the creation of rat periodontal defects to assess in vivo osteogenic ability under pathological conditions. b) Representative micro‐CT images of infected rat periodontal defects at 4 weeks post‐implantation. Red dotted lines denote the boundary between nascent bone and host bone. c) Quantitative analysis of bone volume/ tissue volume (BV/TV) at 4 weeks post‐implantation (*n* = 7). d) Quantitative analysis of bone mineral density (BMD) at 4 weeks post‐implantation (*n* = 7). e) Representative micro‐CT images of infected rat periodontal defects at 8 weeks post‐implantation. Red dotted lines denote the boundary between nascent bone and host bone. f) Quantitative analysis of bone volume/ tissue volume (BV/TV) at 8 weeks post‐implantation (*n* = 7). g) Quantitative analysis of bone mineral density (BMD) at 8 weeks post‐implantation (*n* = 7). h) Representative micro‐CT images of infected rat periodontal defects at 12 weeks post‐implantation. Red dotted lines denote the boundary between nascent bone and host bone. i) Quantitative analysis of bone volume/ tissue volume (BV/TV) at 12 weeks post‐implantation (*n* = 7). j) Quantitative analysis of bone mineral density (BMD) at 12 weeks post‐implantation (*n* = 7). (ns, not significant; ^*^ *p* < 0.05, ^**^ *p* < 0.01, ^***^ *p* < 0.001 and ^****^ *p* < 0.0001).

To achieve complete osseous tissue regeneration, the implantation time was extended to 12 weeks. The micro‐CT results showed that the newly‐formed jaw bone in the HCS‐SF group was similar to the natural jaw bone, without an obvious boundary between the defect area and tooth area (Figure 6b). In marked contrast, abundant high‐density undegraded materials were observed in the Bio group (Figure 6b). The results presented in Figure 6c–f indicated that the bone volume, mean thickness, and coverage of newly‐formed bone were significantly greater in the HCS‐SF group than in the other groups and close to complete healing at 12 weeks post‐implantation. The microstructure of the regenerated osseous tissues after 12 weeks post‐implantation was further evaluated with H&E and Masson's trichrome staining (Figure 6b). The results showed that HCS‐SF implantation led to complete healing with contiguous bone‐structure formation at bone maturity without any hydrogel residue. In contrast, in the Bio groups, a small amount of newly‐formed bone was observed, but there were still a lot of Bio‐Oss residues. Masson's trichrome staining showed that the mature bone in the defect area of the HCS‐SF group was more than that in the Bio and Blank groups. These results thus demonstrated that complete regeneration of the jaw bone could be achieved through HCS‐SF integrated osteoconductive Janus hydrogel treatment. Furthermore, the canonical osteogenic differentiation marker OCN, vascular differentiation marker CD31, and early neural differentiation marker PAX6, were all detectable by immunocytochemical staining at 4 weeks post‐implantation, with the confocal microscopy images indicating that HCS‐SF promoted osteogenic differentiation, vascular differentiation, and neural differentiation at the same time (Figure S6f–i, Supporting Information). Additionally, as compared with the control groups, OCN and the mature neural differentiation marker NF200, were highly expressed in the HCS‐SF group at 12 weeks post‐implantation (Figure 6g–i), which validated mineralization of bone extracellular matrix and neural maturation respectively. Numerous studies have been conducted to develop a diverse array of materials for facilitating bone defect repair, which can induce bone regeneration to varying degrees. Compared with the performance of previous implant materials such as hydrogels and scaffolds, HCS‐SF exhibited higher osteogenic efficiency in the mouse calvarial defect model and in the rat periodontal defect model, increasing by 42% and 13.7% respectively, thus demonstrating that the integrated osteoconductive Janus hydrogel can achieve synergistic osteogenic effects for clinical applications under normal conditions. (Figure 6j; Table S1, Supporting Information). Additionally, an infected rat periodontal defect model was employed to further validate the effects of HCS‐SF on bone regeneration under pathological conditions^[^ ^50^ ^]^ (**Figure** **7**a). After 4 weeks of implantation, the micro‐CT data clearly showed increased mass and improved parameters of new bone formation in the HCS‐SF versus Blank group (Figure 7b–d). Notably, the regenerated bone within the Blank group of the infected periodontal defect model was much less than that in the normal healthy model, confirming that the defect site was indeed infected, which inhibited bone regeneration (Figure 7e–j). In sharp contrast, the amount of newly formed bone in the HCS‐SF group of the infected periodontal defect model was at the same level as that in the normal healthy model, suggesting that HCS‐SF can effectively impede bacterial infection and provide a conducive microenvironment for bone regeneration (Figure S7a–f, Supporting Information). Moreover, SF and CS used in the preparation of the integrated osteoconductive Janus hydrogel are biocompatible and have been approved by FDA for biomedical applications. Hence, these results demonstrated that the integrated osteoconductive Janus hydrogel is an effective implant biomaterial with broad clinical prospects for facilitating craniomaxillofacial bone repair due to its technical simplicity and convenience of application.

### 2.7 The Integrated Osteoconductive Janus Hydrogel Promotes Osteogenesis by Regulating Lipid Metabolism

**Figure 8:**
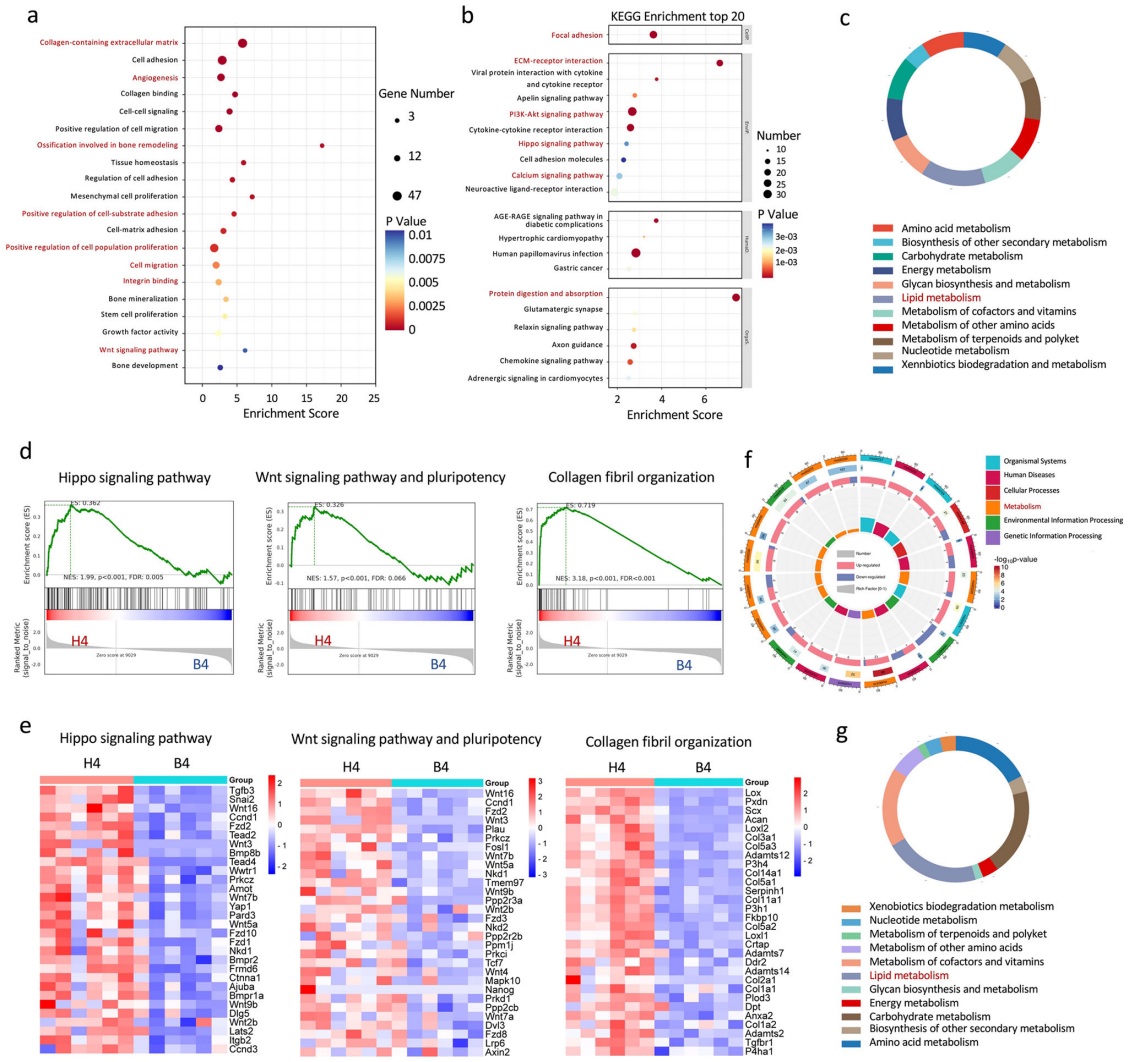
The integrated osteoconductive Janus hydrogel promoted osteogenesis at 4 weeks post‐implantation by regulating lipid metabolism. a) The enriched GO terms of upregulated genes in the HCS‐SF group compared with the Blank group. The red spectrum denotes osteogenesis‐related biological processes. b) Representative KEGG pathways of significant DEGs from the HCS‐SF group versus the Blank group (p ≤ 0.05, |log fold change| ≥1). c) Enrichment of metabolically related KEGG pathways by RNA‐seq analysis. d) GSEA analysis of significant DEGs in the HCS‐SF and Blank groups. e) Gene cluster analysis of osteogenesis‐related genes in the HCS‐SF and Blank groups. f) Circle plot of differential metabolite by KEGG enrichment analysis. g) Circle plot of metabolism‐related pathways by KEGG enrichment analysis.

To further elucidate the the underlying mechanisms driving osteogenesis within the HCS‐SF integrated osteoconductive Janus hydrogel upon implantation in situ, we collected healing samples from the rat periodontal defect for RNA‐seq analysis, at 1 and 4 weeks post‐implantation. As expected, we found that many genes associated with tissue regeneration were upregulated in the differential gene expression volcano map and heat map (Figures S8a,b and S9a,b, Supporting Information). The Gene Ontology (GO) enrichment analysis and the Kyoto Encyclopedia Genes and Genomes (KEGG) enrichment analysis of the functions of differentially expressed genes indicated that the HCS‐SF mainly had an impact on lipid metabolism and tissue regeneration‐related pathways, such as “extracellular matrix organization”, “positive regulation of MSC proliferation” and “ECM‐receptor interaction” at 1 week post‐implantation, and “collagen‐containing extracellular matrix”, “PI3K‐Akt signaling pathway” and “Hippo signaling pathway” at 4 weeks post‐implantation (Figure S8c–e, Supporting Information; **Figure** **8**a–c). The GSEA analysis of the transcriptional results at 1 week post‐implantation mainly focused on cell proliferation and protein synthesis (Figure S8f, Supporting Information). The gene expression heatmap showed that the levels of Col8a1, Itgb7, and Comp were enhanced after 1 week of treatment with HCS‐SF (Figure S8g, Supporting Information). Moreover, the GSEA of the RNA‐Seq results revealed that genes associated with tissue regeneration including “Wnt signaling pathway and pluripotency” and “collagen fibril organization” were enriched in the HCS‐SF group at 4 weeks post‐implantation (Figure 8d). The gene expression heatmap showed that the mRNA levels of Wnt3, Bmpr2, and Yap1 were increased after 4‐weeks of treatment. These signaling pathways are crucial for regulating cell proliferation, differentiation, and migration, which together constituted a complex cascade of biological processes involved in tissue repair and regeneration.

In addition, RNA‐seq analysis suggested a role for metabolic processes in the mechanism of bone regeneration promoted by HCS‐SF. To further explore the characteristics of metabolites in this process, we used LC‐MS/MS technology to analyze various metabolic components and enrichment pathways. Clustered heatmaps showed significant differences in metabolite levels between the HCS‐SF and Blank groups at both 1 and 4 weeks post‐implantation (Figures S8h and S9c, Supporting Information). KEGG enrichment analysis of differential metabolite levels showed that metabolic pathways were significantly enriched in the HCS‐SF group (Figure S8i, Supporting Information; Figure 8f). Further analysis of metabolic pathways indicated that lipid metabolism accounted for the largest proportion among all metabolic pathways at both 1 week and 4 weeks post‐implantation (Figure S8j, Supporting Information; Figure 8g), which was consistent with the enrichment results of differentially expressed genes in the RNA‐seq analysis, thus revealing the key role of lipid metabolism in HCS‐SF‐mediated bone regeneration. Previous studies have shown that the regulation of lipid metabolism contributed to osteogenesis by affecting several biological processes, including cellular energy supply, signaling, and cell growth and differentiation.^[^ ^51^, ^52^ ^]^ Therefore, the HCS‐SF integrated osteoconductive Janus hydrogel may promote rBMSCs osteogenic differentiation by modulating lipid metabolism‐related enzyme molecules or transcription factors.

## 3 Conclusion

An integrated osteoconductive Janus hydrogel comprising of a SF‐MA hydrogel phase and a CS‐MA hydrogel phase to promote intensive bone ingrowth was fabricated by sequential photocuring. The SF‐MA hydrogel phase with barrier protection function prevented the infiltration and ingrowth of gingival fibroblasts, and inhibited *S. aureus* and *E.coli* adhesion, thereby providing a relatively closed biological environment for bone regeneration. Additionally, the CS‐MA hydrogel phase orchestrated lipid metabolism and thus enhanced osteogenesis, angiogenesis, and neurogenesis, thereby enhancing extensive bone regeneration, with osteogenic efficiencies of more than 60% in both the mouse calvarial defect and rat periodontal defect models. Furthermore, this integrated osteoconductive Janus hydrogel achieved adaptive material degradation and facilitated new bone formation with considerable mechanical strength and early structural stability in vivo, which might provide an innovative and well‐suited strategy for bone regeneration therapies. Hence, by combining the advantages of stability, applicability, and simple fabrication techniques, the integrated osteoconductive Janus hydrogel has much promising potential to provide an innovative and well‐suited strategy for bone repair, thereby maximizing therapeutic efficacy in promoting osteogenesis.

## 4 Experimental Section

### Synthesis of Chondroitin Sulfate‐Methacrylate (CS‐MA)

CS was dissolved in deionized water. After full dissolution, the methacrylic anhydride (MA) was added dropwise into the CS solution. The molar ratio of MA versus the hydroxyl groups of CS was 20‐fold. Then, NaOH solution was carefully added to adjust the pH to ≈8. The reaction solution was stirred in an ice bath for 24 h. After the reaction period, the reaction mixture was subjected to dialysis against deionized water to remove the remaining unreacted MA and any by‐products. The CS‐MA was then lyophilized and stored at −20 °C.

### Synthesis of Silk‐Methacrylate (SF‐MA)

SF‐MA solutions were prepared as previously described. Briefly, 5 g of sliced cocoons were boiled in 1 L of 0.02 m Na_2_CO_3_ solution for 30 min at 100 °C to remove the sericin, and then washed and stirred for 20 min with distilled water several times. Subsequently, the degummed silk was dried at room temperature and then dissolved in 5 mL of 4.03 g lithium bromide (LiBr) solution at 60 °C for 1 h. Immediately after the SF was dissolved by LiBr, 0.3 mL (424 mm) of glycidyl methacrylate (GMA) solution (Sigma–Aldrich, St. Louis, USA) was added to the mixture with stirring at 300 rpm for 3 h at 60 °C to create a high‐yield reaction between GMA and SF. Then, the resulting solution was centrifuged and dialyzed against distilled water using Slide‐A‐Lyzer dialysis cassettes (MWCOs of 14000 Da for methacrylated SF solutions) for 3 days. Finally, those solutions were frozen at ‐20 °C for 12 h and freeze‐dried for 48 h. The lyophilized SF‐MA powder was stored at −20 °C before further use.

### Fabrication of the Integrated Osteoconductive Janus Hydrogel

Photo‐curable SF‐MA hydrogels were fabricated as follows. Lyophilized SF‐MA was dissolved in deionized water at a concentration of 30% (w/v), and the photoinitiator lithium phenyl(2,4,6‐trimethylbenzoyl) phosphinate (LAP) (0.2% w/v) (Tokyo Chemical Industry, Tokyo, Japan) was added and mixed. The mixed solution was kept at 4 °C overnight to ensure complete dissolution. The fabrication of photocurable CS‐MA hydrogels was prepared as follows. Lyophilized CS‐MA was dissolved in deionized water and formulated at concentrations of 5% (w/v) and 10% (w/v), and the LAP (0.2% w/v) (Tokyo Chemical Industry, Tokyo, Japan) was added and mixed. The mixed solution was then kept at 4 °C overnight for full dissolution. After the material was completely dissolved, 250 µL of CS‐MA was added into a mold with a diameter of 10 mm, and then subjected to photocuring via exposure to 30 mW cm^−2^ UV light for 5 s at a distance of 1 cm using Architect SV003 (Regenovo). Then 100 µL of SF‐MA was added, and then photocured via exposure to 30 mW cm^−2^ UV light for 30 s at a distance of 1 cm using Architect SV003 (Regenovo), to obtain the integrated osteoconductive Janus hydrogel.

### Characterization of the Integrated Structure

To determine the molecular structure, CS‐MA and SF‐MA were examined through ^1^H nuclear magnetic resonance (^1^H‐NMR) at a frequency of 400 MHz using a Bruker DPX FT‐NMR spectrometer (9.4 T, Bruker, Germany) and 700 µL of deuterium oxide (D_2_O, Sigma‐Aldrich) as the solvent per 5 mg of sample. The SF‐MA solution was filtered using a 0.45 µm filter before analysis. To assess the injectability and photocuring property of the hydrogel, different dyes were added into the precursor solution of CS‐MA and SF‐MA and then the solution was added to syringes and extruded through 26‐gauge needles (φ ≈ 260 µm) to observe the injectability of the hydrogel. The hydrogel was photocured via exposure to UV light for 30 s at a distance of 1 cm respectively. The gelation state and the fabrication of the integrated osteoconductive Janus structure were observed and imaged with a digital camera (Canon camera). To compare the microstructure and pore characteristics among the different hydrogels, field emission scanning electron microscopy (FE‐SEM, S‐4800, HITACHI) was performed after the samples were embrittled with liquid nitrogen, lyophilized, and gold‐coated. For further observation of the interface structure, the CS‐MA solution and SF‐MA solution were mixed with fluorescent dyes of different colors, and the fabricated integrated osteoconductive Janus hydrogel was examined with CLSM (Leica).

### Mechanical Behavior, Swelling and Degradation of the Integrated Osteoconductive Janus Hydrogel

Mechanical properties of these cylindrical hydrogels with dimensions of 5 mm in height and 10 mm in diameter were assessed using a universal mechanical testing machine (WDW3020, China) with a 1 kN load cell at a cross‐head speed of 5 mm min^−1^. Compressive strength and compressive modulus were determined from stress‐strain curves. Compressive strength was the stress at which the sample breaks or the stress at a strain of 90% for highly ductile samples, which do not break until very high strains. Compressive modulus was determined as the slope of the stress‐strain curve within the initial linear region at low strains (0–10%). Otherwise, the dynamic mechanical properties of different hydrogel systems were assessed with a rheometer (TA Instruments‐waters LLC), and all measurement plates were used with a constant gap (1 mm) at room temperature. The loss modulus represents the viscous capacities of the gel, and the resistance of the substance against deformation under shear was measured by the elastic modulus. Cylindrical hydrogels were individually weighted (S_0_) and incubated in PBS solution at 37 °C for 24 h. At predetermined intervals, the hydrogels were carefully taken out, and the residual water on the hydrogel surface was drained with filter papers and weighed (S_1_). The swelling percentage (SP) was determined with Equation (1).

(1) $$\mathrm{SP}=\frac{S1}{S0}\;\times 100\%$$

The in vitro degradation of hydrogels was evaluated in a modified simulated body (SBF) solution (pH = 7.4) at 37 °C for 35 days. The SBF solution was refreshed daily. At predetermined intervals, the residual hydrogels were taken out from the solution, carefully washed with deionized water, and weighed. The weight remaining ratio (WRR) was determined with Equation (2).

(2) $$\mathrm{WRR}=\frac{Wt}{W0}\times 100\%$$

Where W_0_ and W_t_ are the weights of samples before and after degradation for a specific duration time (t) respectively. To assess the in vivo degradation rate of the hydrogel, the CS‐MA and SF‐MA labeled with fluorescent protein were injected into the 3 mm diameter calvarial defect of C57BL/6J mice. The in vivo degradation of the hydrogel was tracked and quantified by the in vivo image system (PerkinElmer).

### Bacterial Adhesion‐Related Surface Property Tests

To further examine the properties related to bacterial adhesion, SF‐MA hydrogels were subjected to water contact angle and surface zeta potential analyses. The water contact angle was measured by a Kruss DSC100 (Germany) instrument via a drop of 2 µL on each hydrogel. Surface zeta potential was measured by a ZETASIZE NANO instrument. The samples were dispersed in ultrapure water and then filtered through a filter. The filtered samples were injected into a polystyrene cuvette and the ZETASIZE NANO instrument automatically calculated the zeta potential for electrophoretic migration.

### Protective Barrier Function Assessment of the SF‐MA Hydrogel

The Gram‐positive bacteria (*S. aureus*, ATCC 25923) and Gram‐negative bacteria (*E.coli*, ATCC 25922) were cultured on SF‐MA hydrogel at 37 °C for 12 and 24 h respectively. To evaluate bacterial adhesion and antibacterial activity, the bacteria were stained with SYT09 and PI fluorescent staining solution (ThermoFisher Scientific) after co‐culture for 15 min, followed by imaging with CLSM (Leica). MTT assay was performed according to the manufacturer's protocol (Solarbio) for the detection of bacterial proliferation. Quantitative analysis of biofilm formation was carried out by crystal violet (Solarbio) staining. Briefly, the samples were washed with PBS, stained with 0.1% (w/v) crystal violet for 20 min, and then washed with deionized water. The crystal violet granules were eluted with 33% (v/v) acetic acid and the absorbance at 595 nm was measured. To evaluate the barrier function of the SF‐MA hydrogel against HGFs, 20 µL aliquots of HGF suspension were seeded onto the surface of each sample at each time point. After culturing for 1, 7, and 14 days, cells on the hydrogels were stained with 4′,6‐diamidino‐2‐phenylindole (DAPI; Sigma) and Phalloidin (Sigma), and observed under CLSM.

### Quantitative Real‐Time Polymerase Chain Reaction (RT‐PCR)

To investigate cell osteogenic differentiation on the integrated osteoconductive Janus hydrogel in vitro, rBMSCs were cultured three‐dimensionally onto the CS‐MA hydrogel for 3 and 7 days separately. Quantitative RT‐PCR was applied to evaluate the expression of osteogenic differentiation gene markers (BMP‐2, Runx2, Col1a1, and OCN). Total RNA was extracted with Trizol reagent (Invitrogen) and synthesis of cDNA was performed using SuperScript III One‐Step RT‐PCR System with Platinum Taq High Fidelity (Invitrogen). Quantitative RT‐PCR was performed on a 7500HT Fast Real‐Time PCR using SYBR Green (Invitrogen). The primer sequences utilized for RT‐PCR were as follows:

**Table:** 

Primers	Forward	Reverse
Rat‐*Gapdh*	TCTCTGCTCCTCCCTGTTC	ACACCGACCTTCACCATCT
Rat‐*Runx‐2*	CTTCCCAAAGCCAGAGCG	CAGCGTCAACACCATCATTC
Rat‐*Bmp‐2*	GAAGCCAGGTGTCTCCA	AGTCCACATACAAAGGGTG
Rat‐*Col1a1*	AGGCAACAGTCGATTCACC	GTCCAAGGGAGCCACATC
Rat‐*Ocn*	AGTCTGACAAAGCCTTC	AAGCAGGGTTAAGCTCAC

### Biocompatibility Assessment

The prepared hydrogels were placed in the wells of standard 96‐well culture plates, and 2 × 10^4^ rBMSCs were seeded per well with LCS, HCS or GelMA, and 2 × 10^4^ HGFs were seeded per well with SF‐MA after ethanol/UV sterilization, and allowed to grow for 14 days. Hydrogel cytocompatibility was analyzed using a Cell Counting Kit 8 (CCK‐8) assay, following the manufacturer's instructions (Bimake). The 96‐well plate was incubated at 37 °C for 20 min in a 5% CO_2_ incubator.

### Immunocytochemistry

To investigate the biological effects of the CS‐MA hydrogel, CS‐MA was dissolved and sterilized through a 0.45 µm filter. A condensed cell suspension at a density of 2 × 10^6^ rBMSCs was seeded into each sample. Subsequently, the samples were slowly added to micro‐tissue 3D petri dishes (Sigma) and photocured. The samples were cultured in an incubator for 3 and 7 days, followed by rinsing with phosphate‐buffered saline (PBS) and fixation with 4% (w/v) paraformaldehyde for 30 min at room temperature. Then, the samples were permeabilized with 0.1% (w/v) Triton X‐100 (diluted with PBS) for 10 min and then blocked with 3% (v/v) bovine serum albumin (BSA; diluted with PBS) for 1 h at room temperature. The permeabilization solution was removed and the samples were rinsed with PBS for 5 min at room temperature. The samples were then incubated respectively with the following primary antibodies in 5% (w/v) BSA in PBS overnight at 4 °C: polyclonal rabbit anti‐RUNX‐2 (1:100; Abcam), polyclonal rabbit anti‐BMP2 (1:200; Abcam). After thorough rinsing to remove excess antibodies, the cells were incubated with the following secondary antibodies for 1 h in the dark: goat anti‐rabbit IgG H&L Alexa Fluor 488 pre‐adsorbed (1:500; Abcam). Phalloidin (Sigma) was used for cytoskeletal staining. Cell nuclei were stained using 4′,6‐diamidino‐2‐phenylindole (DAPI; Sigma). Images were captured using CLSM (Leica). To investigate the barrier effects of the SF‐MA hydrogel, SF‐MA and GelMA were dissolved separately, and sterilized through a 0.45 µm filter. GelMA and SF‐MA were added into micro‐tissue 3D petri dishes, and a sandwich structure of GelMA‐SF‐MA‐GelMA, as well as a simple SF‐MA monolayer structure, were fabricated, which were then subjected to layered photocuring. A condensed cell suspension at a density of 2 × 10^4^ human gingival fibroblasts was seeded into each sample and the samples were cultured in an incubator for 1, 7, and 14 days. The samples were rinsed with phosphate‐buffered saline (PBS) and fixed in 4% (w/v) paraformaldehyde for 30 min at room temperature. Then, the samples were permeabilized with 0.1% (w/v) Triton X‐100 (diluted with PBS) for 10 min and blocked with 3% (w/v) bovine serum albumin (BSA; diluted with PBS) for 1 h at room temperature. The permeabilization solution was removed and the samples were rinsed with PBS for 5 min at room temperature. Blocking with 3% (wv) BSA was used for minimizing non‐specific staining. Phalloidin (Sigma) was used for cytoskeletal staining, while cell nuclei were stained using DAPI (Sigma). Images were captured with CLSM (Leica).

### Western Blotting

Briefly, cell lysate proteins were harvested by RIPA Buffer (Thermo Fisher Scientific), separated by 10% (w/v) SDS–polyacrylamide gel electrophoresis, and then transferred to polyvinylidene difluoride membranes and blocked in 5% (w/v) non‐fat milk. The blotted membranes were separately probed with corresponding primary antibodies against GAPDH (1:5000, RayAntibody), RUNX‐2 (1:1000, Abcam), BMP‐2 (1:500, Affinity), or OCN (1:1000, Abcam) overnight at 4 °C. Then the blotted membranes were washed three times in TBS with 0.1% (v/v) Tween‐20, incubated with a HRP‐conjugated secondary antibody for 1 h, and imaged with an Odyssey Imaging System. Quantitative analysis was performed with the Image J software.

### Animals and Surgical Procedures

The experimental protocol was approved by the Animal Care and Use Committee of Peking University (IACUC number: LA2022604). All the Sprague‐Dawley rats (280–310 g) were randomly divided into three groups: HCS‐SF hydrogel (HCS‐SF), Bio‐Oss+Bio‐Gide (Bio), and Blank (n = 5 for each group). After anesthesia via intravenous injection of 1% (w/v) pentobarbital sodium (1 mg kg^−1^), a 3 mm diameter periodontal defect was created using a saline‐cooled trephine drill and a 3 mm outer diameter treble. Then, the materials of each group were implanted into the defects and the CS‐MA hydrogel and SF‐MA hydrogel were photocured with UV light for 5 and 30 s respectively. The wound was closed by suturing the muscle and the skin layer by layer in the normal healthy model, while those in the infected model were sutured after inoculation with *S. aureus* suspension (2 µL, 10^7^ CFU·mL^−1^) was added onto the surface of the SF‐MA hydrogel. For establishing the calvarial defect model, all the C57BL/6J mice (20–25 g) were randomly divided into five groups: HCS‐SF hydrogel (HCS‐SF), LCS‐SF hydrogel (LCS‐SF), Bio‐Oss+Bio‐Gide (Bio), GelMA, and Blank (n = 5 for each group). The mice were anesthetized via intravenous injection of 1% (w/v) pentobarbital sodium (1 mg kg^−1^) and then two bone defects (3 mm diameter) were prepared in each mouse. Materials were randomly injected into each calvarial defect and then photocured.

### Micro‐CT Scanning Evaluation

At 4, 8, and 12 weeks postimplantation, mandible samples and calvaria samples were harvested and fixed in 4% (w/v) paraformaldehyde for 24 h at 4 °C, and the specimens were examined using micro‐CT scanning. After 3D visualization, bone morphometric analyses, including calculation of BV/TV and BMD measurements, were carried out on the region of interest.

### Histological Analysis

Briefly, tissue samples were fixed in 10% (w/v) neutral buffered formalin for 7 days, decalcified and dehydrated according to standard protocols, embedded in paraffin, and sectioned at 5 µm thickness. H&E staining and Masson's trichrome staining were performed separately on tissue sections, according to the manufacturer's protocols, and images were captured under a light microscope (CX21, Olympus, Japan). CD31, OCN, NF200, and PAX6 expression and distribution were observed using immunocytochemical staining.

### RNA Sequencing, LC‐MS/MS Sequencing and Analysis

Periodontal bone defects were created in rats for RNA‐Seq and LC‐MS/MS analysis. The integrated osteoconductive Janus HCS‐SF hydrogel was injected to fill into the defects of the HCS‐SF group. Subsequently, the periodontal bone defects were sampled at 1 and 4 weeks post‐implantation. For the RNA‐seq analysis, total RNA was extracted using the Trizol reagent (Invitrogen, CA, USA) according to the manufacturer's protocol. RNA purity and quantification were evaluated using the NanoDrop 2000 spectrophotometer (Thermo Scientific, USA). The RNA integrity was assessed using the Agilent 2100 Bioanalyzer (Agilent Technologies, Santa Clara, CA, USA). Then the libraries were constructed using the VAHTS Universal V6 RNA‐seq Library Prep Kit according to the manufacturer's instructions. This was followed by sequencing of the libraries on an Illumina Novaseq 6000 platform and 150 bp paired‐end reads were generated. All genes with |log fold change|>1 and a p‐value of <0.05 were selected as DEGs. Based on the hypergeometric distribution, GO, KEGG pathway, Reactome and WikiPathways enrichment analysis of DEGs were performed to screen the significant enriched term using R (v 3.2.0), respectively. The Gene Set Enrichment Analysis (GSEA) was performed using the GSEA software. For the LC‐MS/MS Sequencing, 30 mg of sample was added to a 1.5 mL Eppendorf tube with 400 µL of methanol‐water (V:V = 4:1) as internal standard. The mixtures were stored at −40 °C for 2 min and placed in a grinder (60 Hz, 2 min). The whole samples were extracted by ultrasonic treatment for 10 min in an ice‐water bath, and stored at −40 °C for 120 min. The extract was then centrifuged at 4 °C (13000 rpm) for 20 min. The supernatants (150 µL) from each tube were collected using crystal syringes and transferred to LC vials. The vials were stored at −80 °C until LC‐MS analysis. QC samples were prepared by mixing aliquots of the samples to form a pooled sample. The metabolomic data analytical instrument utilized was the ACQUITY UPLC I‐Class plus instrument (Waters Corporation, Milford, USA). The original LC‐MS data were processed by the Progenesis QI V2.3 (Nonlinear, Dynamics, Newcastle, UK) software for baseline filtering, peak identification, integration, retention time correction, peak alignment, and normalization. A two‐tailed Student's T‐test was further used to verify whether the differential metabolite levels between groups were significantly different. Differential metabolites were selected with p‐values less than 0.05 and were further used for KEGG pathway enrichment analysis.

### Statistical Analysis

All quantitative data were expressed as mean ± standard deviation (SD) of at least three independent replicate experiments. The Shapiro‐Wilk test was used to assess the normality of distributions. Statistical analyses were carried out using One‐Way analysis of variance (ANOVA) combined with the Student‐Newman‐Keuls (SNK) multiple comparison post‐hoc test. The thresholds of statistical significance were set at ^*^ *p* < 0.05, ^**^ *p* < 0.01, ^***^ *p* < 0.001, and ^****^ *p* < 0.0001.

## Conflict of Interest

The authors declare no conflict of interest.

## Supporting information



Supporting Information

## Data Availability

The data that support the findings of this study are available from the corresponding author upon reasonable request.
