# Supplementary material for: An Osteoconductive Janus Hydrogel with Full Barrier Protection and Adaptable Degradation Properties for Superior Bone Regeneration
Source: Adv Sci (Weinh). 2025 Jun 23;12(34):e06736. doi: 10.1002/advs.202506736 (PMC12442636; doi:10.1002/advs.202506736)
Supplement: Supplementary file 1 — Supporting Information [file ADVS-12-e06736-s001.pdf]

ADVANCED SCIENCE
Open Access

Supporting Information

for *Adv. Sci.*, DOI 10.1002/advs.202506736

An Osteoconductive Janus Hydrogel with Full Barrier Protection and Adaptable Degradation Properties for Superior Bone Regeneration

Yanhui Lu, Jia Song, Yongle Lv, Boon Chin Heng, Mingming Xu, Ying He, Youde Liang, Lu-Ning Wang, Tingting Wu, Ting Song, Tingjun Li, Qiaomei Ren, Lei Wang, Xuliang Deng* and Xuehui Zhang**

Supplementary Information

An osteoconductive Janus hydrogel with full barrier protection and adaptable degradation properties for superior bone regeneration

Authors: Yanhui Lu, Jia Song, Yongle Lv, Boon Chin Heng, Mingming Xu, Ying He, Youde Liang, Lu-Ning Wang, Tingting Wu, Ting Song, Tingjun Li, Qiaomei Ren, Lei Wang*, Xuliang Deng*, Xuehui Zhang*

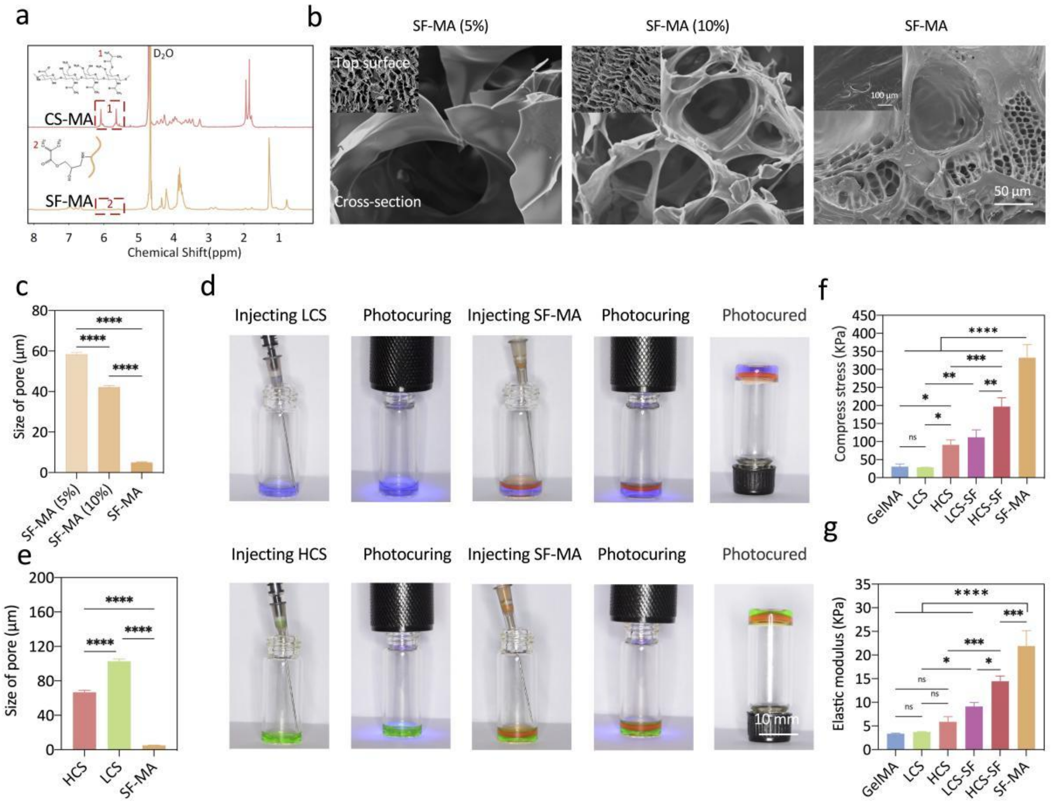

Figure S1. Fabrication and characterization of the osteoconductive Janus hydrogel.

(a) The ^{1}H NMR spectra (400 MHz, D_2O) of SF-MA and CS-MA.

(b) Representative SEM images of the SF-MA hydrogel with mass fractions of 5%, 10% and 30%.

(c) Quantitative analysis of pore sizes of SF-MA hydrogel with mass fractions of 5%, 10% and 30%.

(d) Schematic representation of the construction process of the integrated osteoconductive biphasic hydrogel.

(e) Quantitative analysis of pore sizes within each layer of the integrated osteoconductive biphasic hydrogel.

(f) Quantitative analysis of compressive strength of the integrated osteoconductive biphasic hydrogel.

(g) Quantitative analysis of elastic moduli of the integrated osteoconductive biphasic hydrogel. Error bars represent the standard error of the mean. (ns, not significant; *$p < 0.05$, **$p < 0.01$, ***$p < 0.001$ and ****$p < 0.0001$).

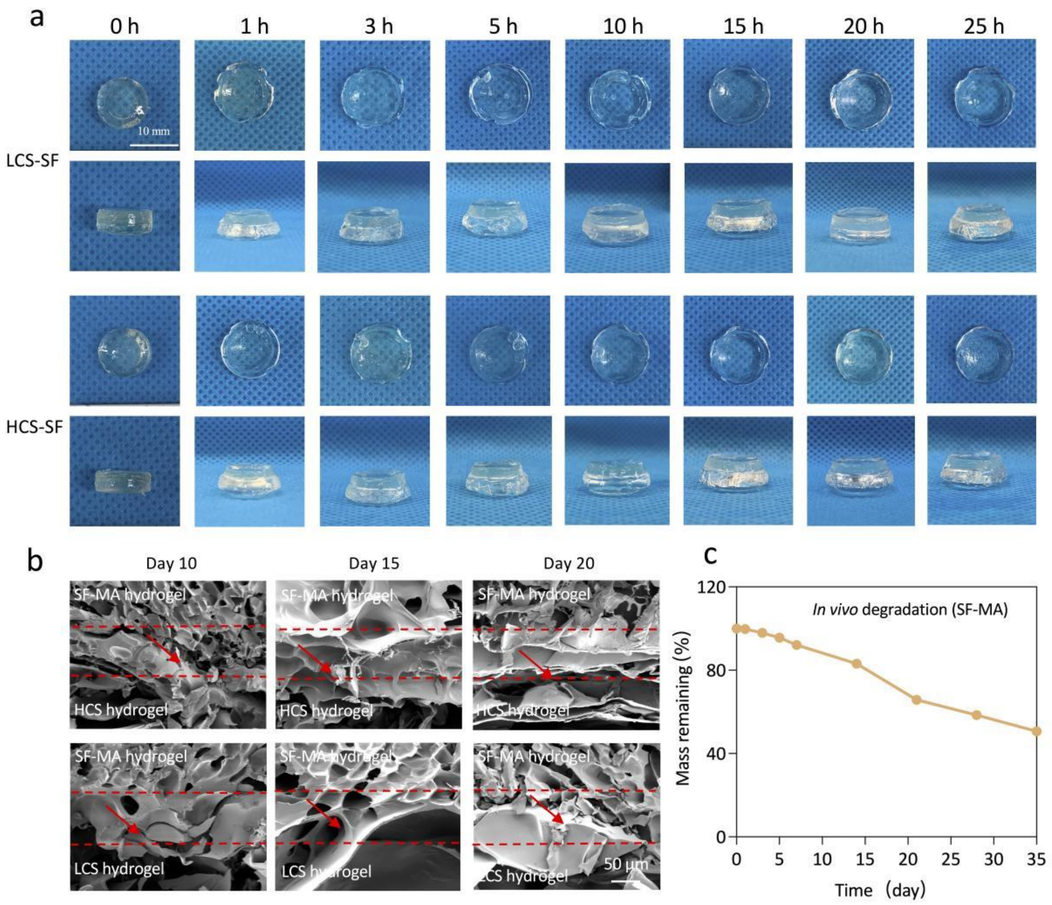

Figure S2. Physico-chemical properties of the osteoconductive Janus hydrogel within a simulated *in vitro* environment to mimic *in vivo* implantation conditions.

(a) Real-time images of the swelling process of the integrated osteoconductive biphasic hydrogel.

(b) Representative SEM images after 10, 15 and 20 days of hydrogel degradation. The area between the red dotted lines denotes the fusion of the CS-MA hydrogel phase with the SF-MA hydrogel phase.

(c) *In vivo* degradation properties of the SF-MA hydrogel. Error bars represent the standard error of the mean. (****$p < 0.0001$).

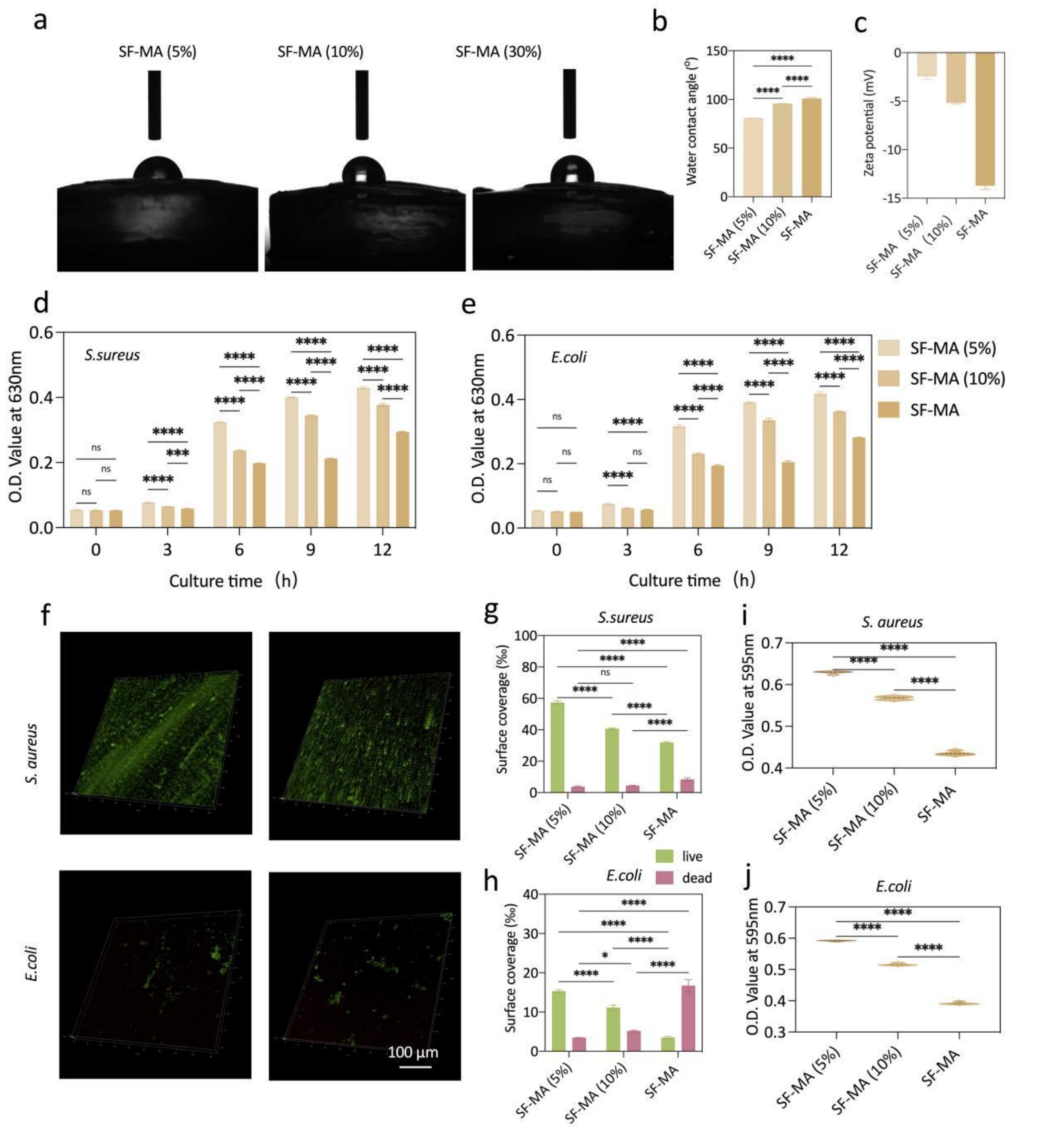

Figure S3. Physico-chemical properties and anti-bacterial adhesion of the SF-MA hydrogel phase within a simulated *in vitro* implantation environment.

(a) Water contact angle images of SF-MA with mass fractions of 5%, 10% and 30%.

(b) Quantitative analysis of water contact angles.

(c) Quantitative analysis of zeta potential values of SF-MA with mass fractions of 5%, 10% and 30%.

(d) Quantitative analysis of O.D. values after 12 h co-culture with *S. aureus.*

(e) Quantitative analysis of O.D. values after 12 h co-culture with *E.coli.*

(f) The representative live & dead staining images of *S. aureus* and *E.coli* after 12 h co-culture with SF-MA with mass fractions of 5% and 10%.

(g) Quantification of the fluorescence area based on the live/dead fluorescence staining images of *S. aureus*.

(h) Quantification of the fluorescence area based on the live/dead fluorescence staining images of *E.coli*.

(i) Semi-quantification of crystal violet staining after 48 h co-culture with *S. aureus*.

(j) Semi-quantification of crystal violet staining after 48 h co-culture with *E.coli*.

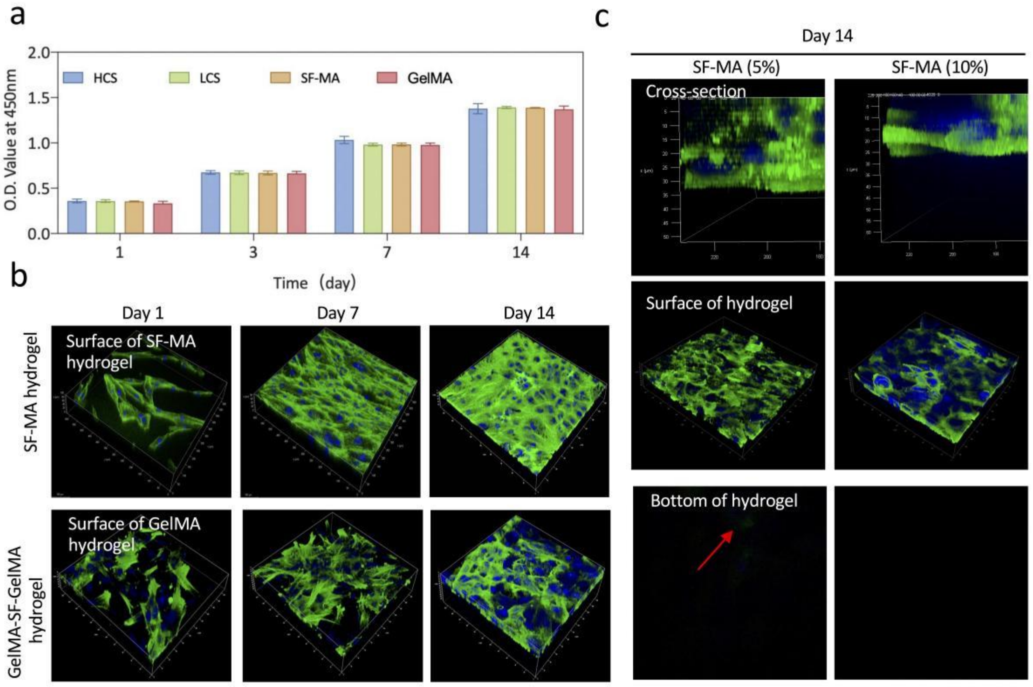

Figure S4 Biocompatibility of the integrated osteoconductive Janus hydrogel and barrier function of the SF-MA hydrogel phase within a simulated *in vitro* environment to mimic *in vivo* implantation conditions.

(a) Biocompatibility assessment of the integrated osteoconductive Janus hydrogel.

(b) Representative immunostaining images of gingival fibroblasts cultured on the SF-MA hydrogel and GelMA-SF-MA-GelMA sandwich structure for 1, 7 and 14 days.

(c) Representative immunostaining images of gingival fibroblasts cultured on SF-MA with mass fractions of 5% and 10% for 14 days.

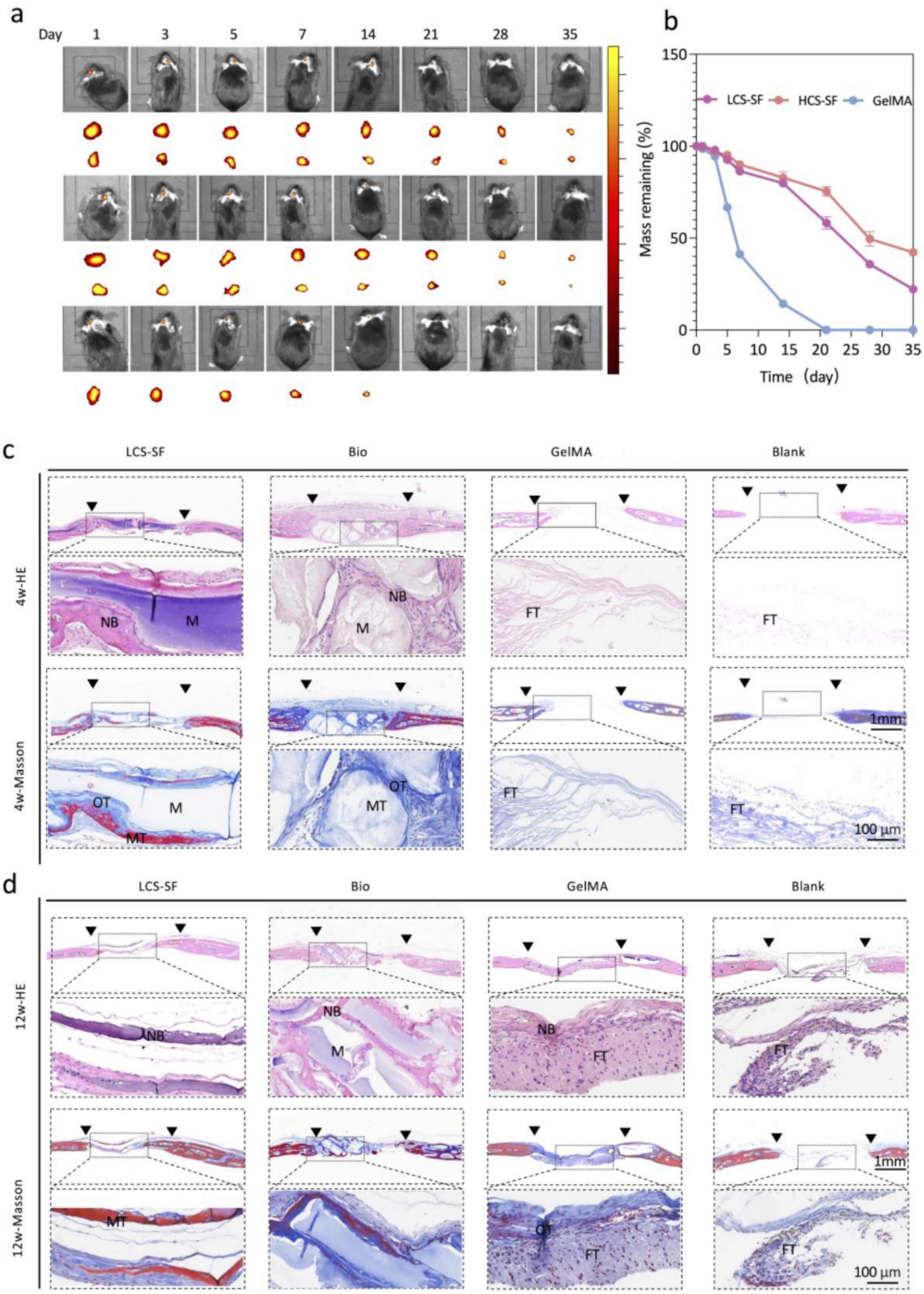

Figure S5. Degradation of scaffold materials and bone formation after implantation of the integrated osteoconductive Janus hydrogel *in vivo*.

(a) Representative degradation images of the integrated osteoconductive biphasic hydrogel *in vivo*.

(b) Quantification of the remaining mass based on the fluorescence area.

(c-d) H&E staining and Masson's trichrome staining of histological sections within the Bio and Blank groups at 4 weeks and 12 weeks after implantation. (FT, fibrous tissue; NB: nascent bone; MT, mineralized tissue; M, residual materials).

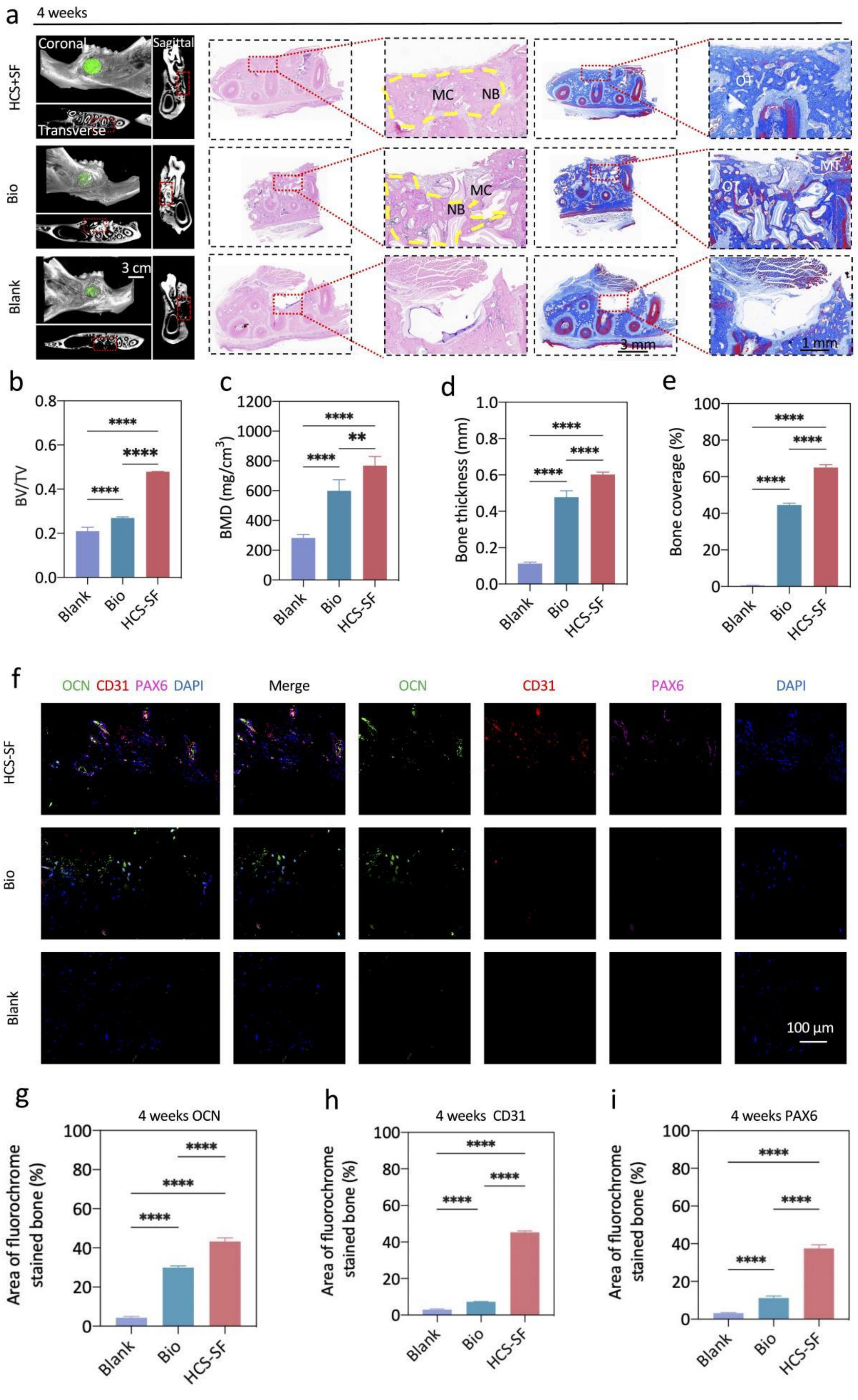

Figure S6. ***In situ*** **osteogenic differentiation of rat mandibular defect with the integrated osteoconductive Janus hydrogel at 4 weeks post-implantation.**

(a) Representative micro-CT images of rat mandibular defects at 4 weeks post-implantation. Red dotted lines denote the boundary between the nascent bone and the host bone. The green fake color denotes the nascent bone. H&E staining and Masson's trichrome staining of histological sections at 4 weeks after implantation. The part circled by the yellow dotted line denotes the newly-formed bone within the defect area. (FT, fibrous tissue; NB: nascent bone; OT, osteoid tissue; MT, mineralized tissue; MC, marrow cavity).

(b-e) Quantitative analysis of bone volume/ tissue volume, bone mineral density (BMD), bone thickness and bone coverage at 4 weeks.

(f) Immunohistochemical staining images of OCN (Green), CD31 (Red) and PAX6 (Pink) from the rat mandible defect after implantation of the integrated bilayer injectable hydrogel for 4 weeks. DAPI stained cell nuclei (Blue).

(g-i) The mean fluorescence intensities were calculated to evaluate protein expression levels. Error bars represent the standard error of the mean. (ns, not significant; *$p < 0.05$, **$p < 0.01$, ***$p < 0.001$ and ****$p < 0.0001$).

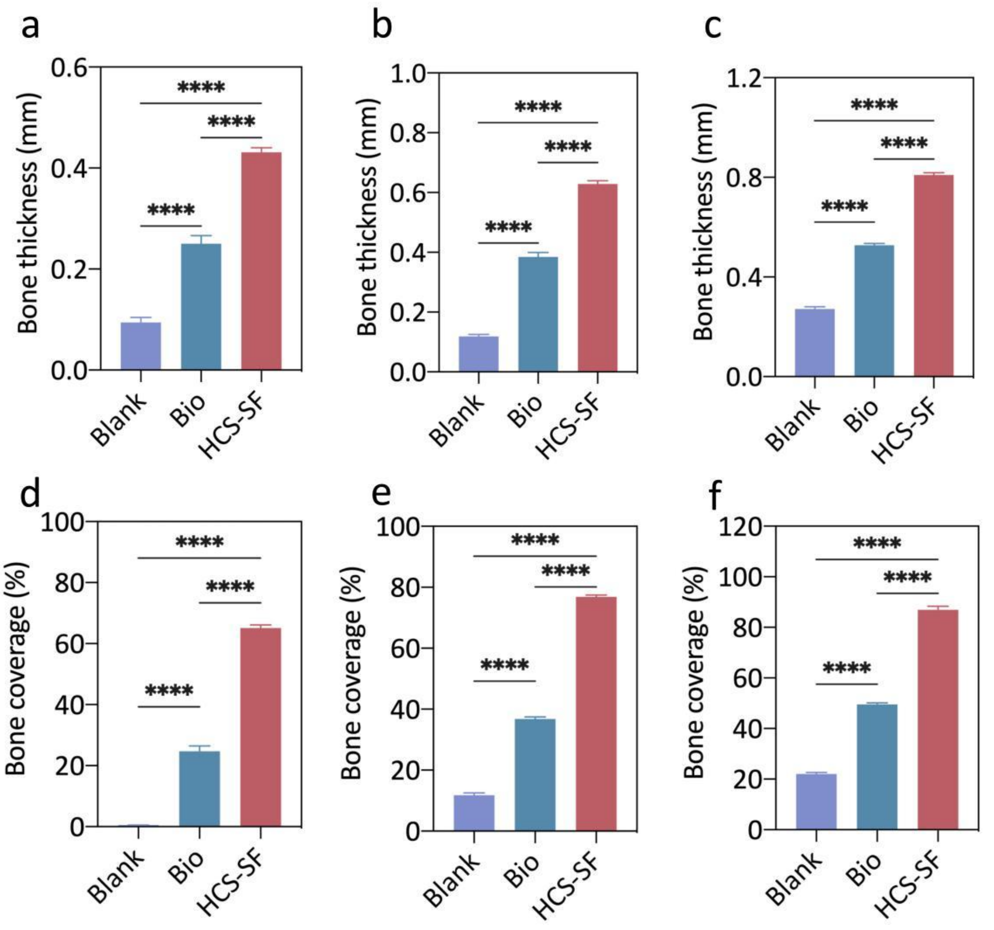

Figure S7. ***In situ*** **osteogenic differentiation of infected rat periodontal defect with the integrated osteoconductive Janus hydrogel.**

(a) Quantitative analysis of bone thickness at 4 weeks post-implantation (n = 7).

(b) Quantitative analysis of bone thickness at 8 weeks post-implantation (n = 7).

(c) Quantitative analysis of bone thickness at 12 weeks post-implantation (n = 7).

(d) Quantitative analysis of bone coverage at 4 weeks post-implantation (n = 7).

(e) Quantitative analysis of bone coverage at 8 weeks post-implantation (n = 7).

(f) Quantitative analysis of bone coverage at 12 weeks post-implantation (n = 7). (ns, not significant; $*p < 0.05$, $**p < 0.01$, $***p < 0.001$ and $****p < 0.0001$).

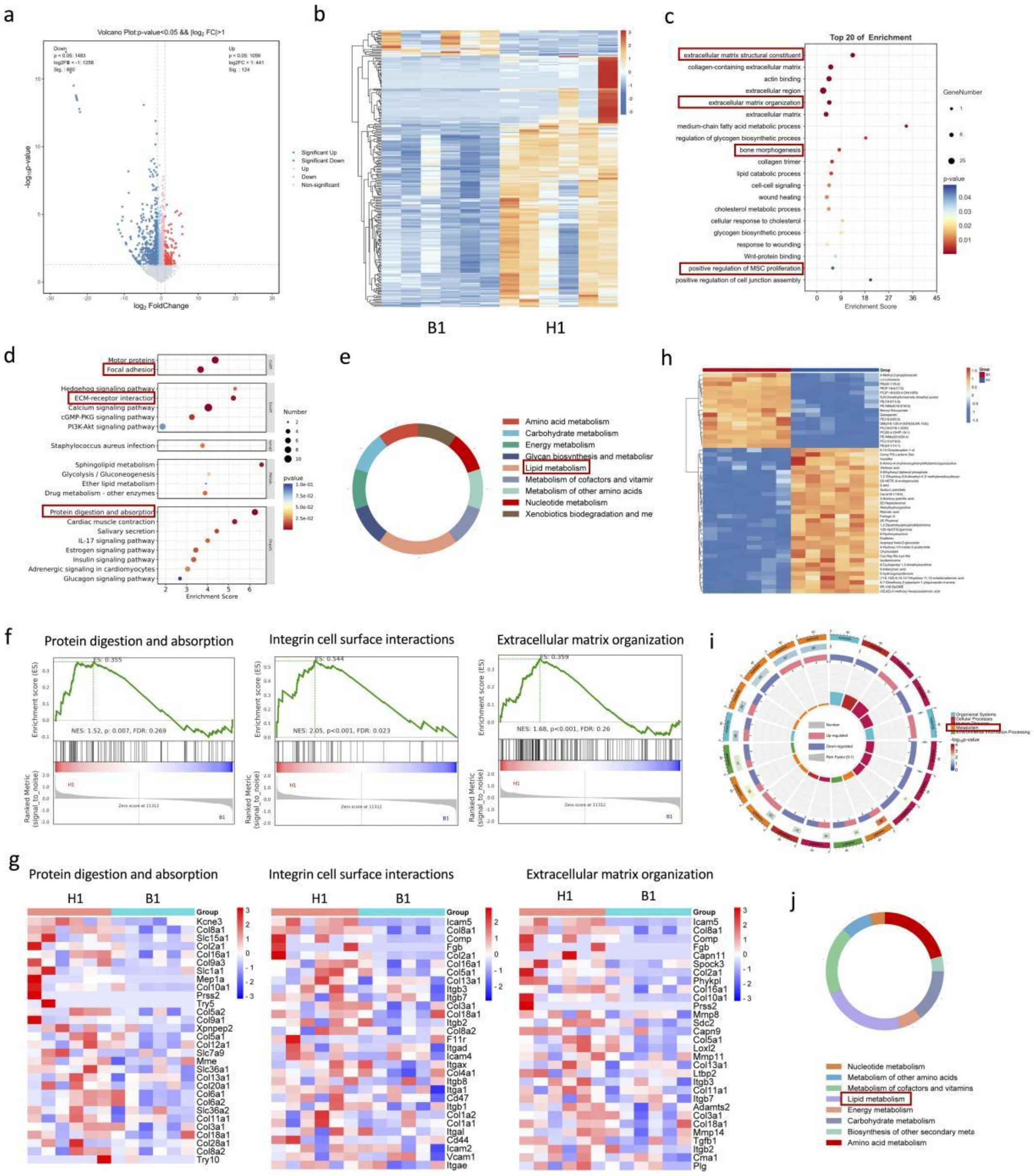

Figure S8. Underlying mechanisms at 1 week post-implantation, as elucidated by RNA-seq and LC-MS/MS analysis.

(a) The volcano plot of differentially-expressed genes (DEGs) in the HCS-SF group compared with the Blank group by RNA-Seq analysis.

(b) The RNA-Seq analysis heat map of DEGs in the HCS-SF group compared with the Blank group. .

(c) The enriched GO terms of upregulated genes in the HCS-SF group compared with the Blank group. The biological processes in the red box denote osteogenesis.

(d) Representative KEGG pathways of significant DEGs from the HCS-SF group versus the Blank group ($p \leq 0.05$, $|\log \text{fold change}| \geq 1$).

(e) Enrichment of metabolically related KEGG pathways by RNA-seq analysis.

(f) GSEA analysis of significant DEGs in HCS-SF and Blank groups.

(g) Gene cluster analysis of osteogenesis-related genes in the HCS-SF and Blank groups.

(h) The heat map of differential metabolites.

(i) Circle plot of differential metabolite by KEGG enrichment analysis.

(j) Circle plot of metabolism-related pathways by KEGG enrichment analysis.

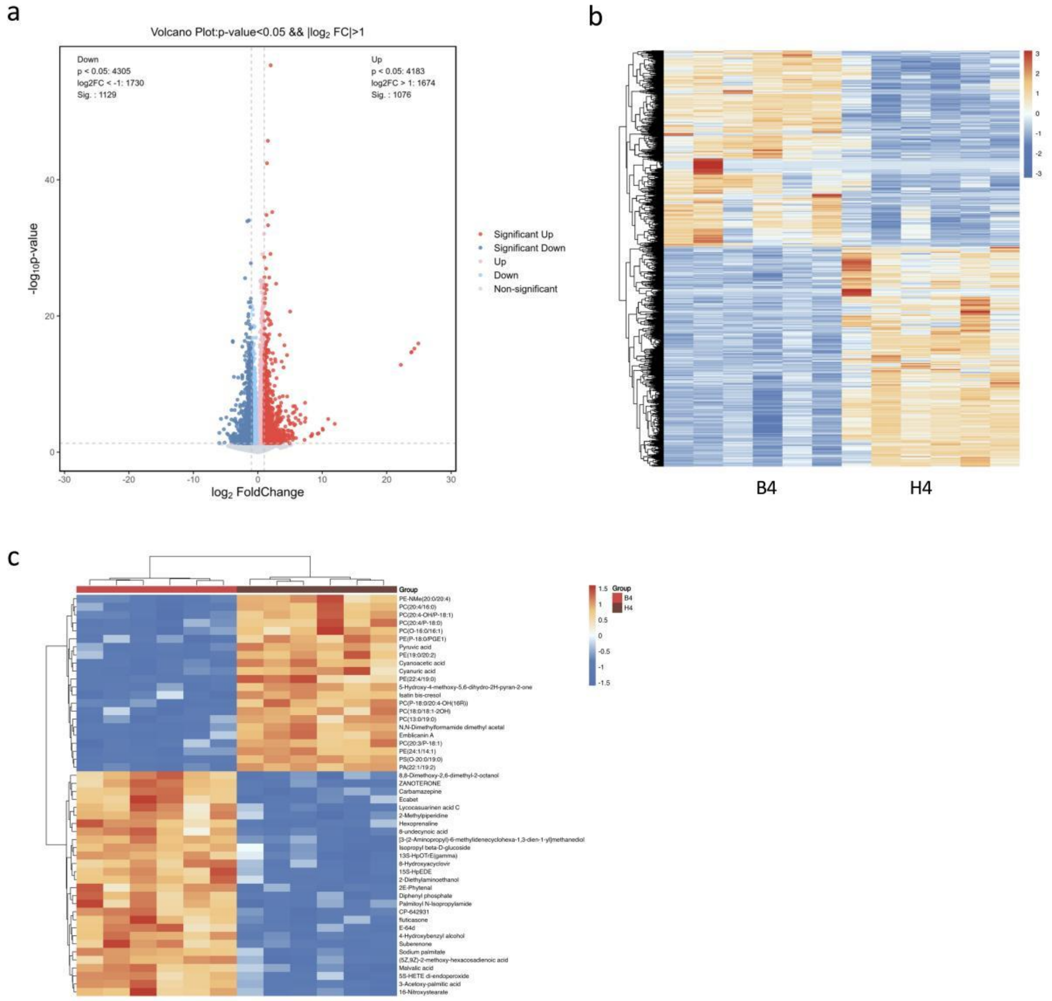

Figure S9. Underlying mechanisms at 4 weeks post-implantation, as elucidated by RNA-seq and LC-MS/MS analysis.

(a) The volcano plot of differentially-expressed genes (DEGs) in the HCS-SF group compared with the Blank group by RNA-Seq analysis.

(b) The heat map of DEGs in the HCS-SF group compared with the Blank group by RNA-Seq analysis.

(c) The heat map of differential metabolites.

Table S1 Osteogenesis efficiency and material morphology of the referenced Materials

Material name	Defect type	Morphology	Data form	Osteogenesis efficiency (%)	References
NoBS with SAG	mouse calvarial defect	hydrogel	BV/TV	30 (8w)	[1]
Scaffold+MSC+NIR	mouse calvarial defect	hydrogel	BV/TV	22 (4w)	[2]
fGelMA+MSC+NIR	mouse calvarial defect	hydrogel	BV/TV	28 (8w)	[2]
EM-OMN/hydrogel	mouse calvarial defect	hydrogel	BV/TV	22 (8w)	[3]
RNA-ACP	mouse calvarial defect	scaffold	BV/TV	18 (4w)	[4]
RNA-ACP	mouse calvarial defect	scaffold	BV/TV	33 (6w)	[4]
At-DNA@S	mouse calvarial defect	hydrogel	BV/TV	22 (4w)	[5]
At-DNA@S	mouse calvarial defect	hydrogel	BV/TV	47 (8w)	[5]
CGG-Cit	mouse calvarial defect	hydrogel	BV/TV	25 (12w)	[6]
BMP2-exosome loaded GelMA hydrogel	mouse calvarial defect	hydrogel	BV/TV	39 (8w)	[7]
HNS	mouse calvarial defect	hydrogel	BV/TV	35(8w)	[8]
Porous scaffold	mouse calvarial defect	hydrogel	BV/TV	36 (8w)	[9]
PFCH nanofibrous membranes	mouse calvarial defect	membrane	BV/TV	14.5 (4w)	[10]
PUR-containing sterosome scaffold	mouse calvarial defect	scaffold	BV/TV	22 (6w)	[11]

SMPU/4wt%Mg	mouse calvarial defect	scaffold	BV/TV	19.8 (12w)	[12]
CGF/IMC	rat periodontal defect	scaffold	BV/TV	37 (4w)	[13]
RVS+SrRn	rat periodontal defect	scaffold	BV/TV	24 (8w)	[14]
IMC/Zno	rat periodontal defect	scaffold	BV/TV	49.53 (6w)	[15]
HIMC	rat periodontal defect	scaffold	BV/TV	24.4 (10w)	[16]
Gelma+5%Mn-SiHANW	rat periodontal defect	scaffold	BV/TV	42 (8w)	[17]
L-Cys-AuNPs	rat periodontal defect	scaffold	BV/TV	38 (3w)	[18]
3DR	rat periodontal defect	scaffold	BV/TV	12.5 (8w)	[19]
CS-sEVs	rat periodontal defect	scaffold	BV/TV	59 (8w)	[20]
PDLLA-PEG-PDLLA-Met@MSN-SDF-1 hydrogel	rat periodontal defect	hydrogel	BV/TV	28 (4w)	[21]
PDLLA-PEG-PDLLA-Met@MSN-SDF-1 hydrogel	rat periodontal defect	hydrogel	BV/TV	54 (8w)	[21]
GPEGD	rat periodontal defect	hydrogel	BV/TV	24.7 (4w)	[22]
GPEGD	rat periodontal defect	hydrogel	BV/TV	41.47 (8w)	[22]
EMP@P.g	rat periodontal defect	hydrogel	BV/TV	50 (12w)	[23]
Bio-GelMA@Bio-Exs	rat periodontal defect	hydrogel	BV/TV	50 (4w)	[24]
DFAT sheet	rat periodontal defect	membrane	BV/TV	58 (8w)	[25]

HCS-SF	mouse calvarial defect	hydrogel	BV/TV	64.4 (12w)	Our work
HCS-SF	rat periodontal defect	hydrogel	BV/TV	63.7 (12w)	Our work

References

1. C. S. Lee, H. S. Hwang, S. Kim, J. Fan, T. Aghaloo, M. Lee, Adv Funct Mater. 2020, 30, 2003717.

2. S. Shen, R. Liu, C. Song, T. Shen, Y. Zhou, J. Guo, B. Kong, Q. Jiang, NANO RES. 2023, 16, 7383-7392.

3. J. Fan, C. S. Lee, S. Kim, C. Chen, T. Aghaloo, M. Lee, ACS Nano 2020, 14, 11973.

4. M. J. Shen, C. Y. Wang, D. X. Hao, J. X. Hao, Y. F. Zhu, X. X. Han, L. Tonggu, J. H. Chen, K. Jiao, F. R. Tay, L. N. Niu, Adv Mater. 2022, 34, e2107924.

5. Y. Han, Y. Wu, F. Wang, G. Li, J. Wang, X. Wu, A. Deng, X. Ren, X. Wang, J. Gao, Z. Shi, L. Bai, J. Su, Bioact Mater. 2024, 35, 1.

6. T. Liu, Z. You, F. Shen, P. Yang, J. Chen, S. Meng, C. Wang, D. Xiong, C. You, Z. Wang, Y. Shi, L. Ye, ACS Appl Mater Interfaces. 2024, 16, 5486.

7. J. Sun, G. Li, S. Wu, Y. Zou, W. Weng, T. Gai, X. Chen, K. Zhang, F. Zhou, X. Wang, COMPOS PART B-ENG. 2023, 261, 110803.

8. S. Hao, D. Zhou, F. Wang, G. Li, A. Deng, X. Ren, X. Wang, Y. Jing, Z. Shi, L. Bai, J. Su, Chem. Eng. J. 2024, 489, 150990.

9. Z. Guo, L. Dong, J. Xia, S. Mi, W. Sun, 3D Printing Unique Nanoclay-Incorporated Double-Network Hydrogels for Construction of Complex Tissue Engineering Scaffolds. Adv Healthc Mater. 2021, 10, e2100036.

10. S. Jin, R. Yang, C. Chu, C. Hu, Q. Zou, Y. Li, Y. Zuo, Y. Man, J. Li, Acta Biomater. 2021, 129, 148.

11. C. S. Lee, S. Kim, J. Fan, H. S. Hwang, T. Aghaloo, M. Lee, Sci Adv. 2020, 6, eaaz7822.

12. Y. Zhang, C. Li, W. Zhang, J. Deng, Y. Nie, X. Du, L. Qin, Y. Lai, Bioact Mater.

2021, 16, 218.
13. M. Yu, D. Luo, J. Qiao, J. Guo, D. He, S. Jin, L. Tang, Y. Wang, X. Shi, J. Mao, S. Cui, Y. Fu, Z. Li, D. Liu, T. Zhang, C. Zhang, Z. Li, Y. Zhou,Y. Liu, Bioact Mater. 2021, 10, 93.
14. W. Zhang, W. Shi, S. Wu, M. Kuss, X. Jiang, J. B. Untrauer, S. P. Reid, B. Duan, Biofabrication. 2020, 12, 035020.
15. Y. Zhang, Z. Li, B. Guo, Q. Wang, L. Chen, L. Zhu, T. Zhang, R. Wang, W. Li, D. Luo, Y. Liu, Small 2024, 20, e2309230.
16. S. S. Jin, D. O. He, D. Luo, Y. Wang, M. Yu, B. Guan, Y. Fu, Z. X. Li, T. Zhang, Y. H. Zhou, C. Y. Wang, Y. Liu, ACS Nano 2019, 13, 6581.
17. R. Li, Z. Zhu, B. Zhang, T. Jiang, C. Zhu, P. Mei, Y. Jin, R. Wang, Y. Li, W. Guo, C. Liu, L. Xia, B. Fang, Adv Sci (Weinh). 2024, 11, e2305890.
18. S. Zhang, H. Zhou, N. Kong, Z. Wang, H. Fu, Y. Zhang, Y. Xiao, W. Yang, F. Yan, Bioact Mater. 2021, 6, 3288.
19. X. Xu, Y. Zhou, K. Zheng, X. Li, L. Li, Y. Xu, ACS Appl Mater Interfaces. 2022, 14, 46145–46160.
20. L. Ma, N. Rao, H. Jiang, Y. Dai, S. Yang, H. Yang, J. Hu, Stem Cell Res Ther. 2022, 13, 92.
21. H. Wang, X. Chang, Q, Ma, B. Sun, H. Li, J. Zhou, Y. Hu, X. Yang, J. Li, X. Chen, J. Song, Bioact Mater. 2022, 21, 324.
22. Y. Wu, X. Li, Y. Sun, X. Tan, C. Wang, Z. Wang, L. Ye, Bioact Mater. 2022, 20, 111.
23. G. T. Yu, W. X. Zhu, Y. Y. Zhao, H. Cui, H. Chen, Y. Chen, T. T. Ning, M. D. Rong, L. Rao, D. D. Ma, Biofabrication. 2024, 16, 025007.
24. D. Deng, X. Li, J. J. Zhang, Y. Yin, Y. Tian, D. Gan, R. Wu, J. Wang, B. M. Tian, F. M. Chen, X. T. He, ACS Nano 2023, 17, 8530.
25. G. Huang, B. Xia, Z. Dai, R. Yang, R. Chen, H. Yang, J Clin Periodontol. 2022, 49, 1289.
